# Exploring the role of ferroptosis in esophageal cancer: mechanisms and therapeutic implications

**DOI:** 10.1038/s41420-025-02696-2

**Published:** 2025-08-25

**Authors:** Defeng Zhao, Wenze Li, Zhongyu Han, Ziyi Wang, Danni Li, Wenya Li

**Affiliations:** 1 Department of Thoracic Surgery, The First Hospital of China Medical University, Shenyang, China; 2 Department of Hematology, The First Hospital of China Medical University, Shenyang, China; 3 Institute of Nephrology, Zhongda Hospital, Southeast University, Nanjing, China; 4 Department of Medical Oncology, The First Hospital of China Medical University, Shenyang, China

**Keywords:** Oesophageal cancer, Cell death, Cell death and immune response

## Abstract

With the development of medical and health care, esophageal cancer (EC) has become a disease of concern to the scientific research community. At present, among all treatment regimens for EC, surgical resection is conducive to the prognosis of early patients neoadjuvant therapies are recommended for advanced patients. However, treatments now are not satisfactory in suppressing the progression of EC. Ferroptosis is one distinctive cell death mode, noted for the accumulation of iron as well as lipotoxicity, which induce cell membrane to breakdown. As a star protein of ferroptosis related pathway, GPX4 is related to the homeostatic imbalance of tumor immune microenvironment (TIME) of EC, thereby regulating the onset as well as progression of the cancer. In our manuscript, we present the mechanisms involved in ferroptosis, the functions of ferroptosis in the TIME. We also focused on the progression about ferroptosis in EC, as well as targeting ferroptosis-related pathways to delay the development of EC. We expect that these contents can expand fresh insights and aim for EC therapeutic strategy in clinical practice.

## FACTS

- Ferroptosis is a distinctive type of regulated cell death driven by iron-dependent lipid peroxidation.
- Ferroptosis functions in conquering tumor resistance thereby become novel strategy for cancer treatment.
- Ferroptosis is active in tumor microenvironment including immune cells, metabolism reprogramming.
- Ferroptosis have shown significant effect in providing new hope to treat esophageal cancer and predict its prognosis.
- Ferroptosis in combination with chemo-/radio-/immuno- and target therapy are proved to have great potential in elevate the clinical outcomes of esophageal patients.

## Introduction

Esophageal cancer (EC) is a common malignancy of the digestive system, with about 604,000 newly identified patients with 544,000 deaths every year [1]. EC is broadly divided into 2 categories: Esophageal adenocarcinoma (EAC) and esophageal squamous cell carcinoma (ESCC) [2]. At present, there are several main methods treating EC, including surgery, neoadjuvant or adjuvant immunotherapy, radiotherapy, target therapy and chemotherapy [3]. However, these means did not show satisfactory effect to terminal tumor. Unfortunately, due to EC’s concealment features, most patients have been found to have advanced tumor already when seeking treatment at a hospital for the first time. A study shows that the median survival in EC patients who are first diagnosed with distant organ metastasis were only 6 months [4]. Thus, there is a pressing need to devise novel effective therapeutic strategies for EC.

Ferroptosis have become research hotspot in cancer treatment in recent years. It is lately recognized as a distinctive type of regulated cell death (RCD) driven by iron-dependent lipid peroxidation [5]. Cells in turns have evolved multiple defense systems which detoxifying lipid peroxides. Ferroptosis may conquer tumor resistance to traditional RCD and become a feasible treating means [6]. It has also shown therapeutic potential and research value in EC. Recent studies revealed that many key pathways and genes of ferroptosis have directly or indirectly involved in the development and prognosis of EC [7–9]. Therefore, focusing on ferroptosis-related genes and pathways could potentially drive additional advancements in EC research.

This manuscript reviews the progress of ferroptosis and its research in EC. We discuss the role of ferroptosis in EC treatment, EC immune microenvironment, and prediction of prognosis in EC patients. We hope this manuscript can bring new means and innovations for the direction of EC treatment.

## Major milestones for ferroptosis

Over the past few years, studies exploring ferroptosis have proliferated remarkably [10]. The term “ferroptosis” was introduced in 2012 [11], yet investigations into this phenomenon began much earlier. The toxicity induced by iron was first observed in 1908 [12]. In 1955, the effects of cystine on survival and proliferation of mice fibroblast L cells and HeLa cells were recorded [13]. Till 1959, it was discovered that cystine and selenium could notably diminish peroxidation levels in the livers and muscles of chickens suffering from vitamin E deficiency [14]. Shiro Bannai showed in 1977 that cell death promoted by cystine was accompanied by consumption of glutathione (GSH), the supplementation of the antioxidant vitamin E prevented this process [15]. They soon mentioned in 1980 that the system xc^-^ could absorb cystine from the environment, thereby acquiring glutamate [15].

Glutathione Peroxidase 4 (GPX4), a selenoprotein, was recognized in the year of 1982 to be one GSH-dependent peroxidase that defends against lipid peroxidation within cell membranes, which an important milestone [16]. Immediately afterwards, it was shown that GPX4 could prevent cell death caused by lipid peroxidation [17]. In 1989, inhibition of cystine uptake was observed to cause glutamate-induced cytotoxicity, resulting in reduced GSH and ultimately cell death [18].

Antioxidant means, such as α-tocopherol as well as ALOX12, can counteract such cell death [19]. In 2001, the term “oxytosis” was coined to refer to neuronal nonapoptotic cell death due to oxidative stress caused by toxic reactions to glutamate [20]. Oxytosis and ferroptosis share some similarities, which include the ability to produce reactive oxygen species (ROS), consume Arachidonate lipoxygenases (ALOXs) as well as GSH. These progresses are inhibited by iron chelators, as well as are intensified by other sources of iron [21]. However, some specific features of oxytosis, such as mitochondrial swelling and DNA fragmentation [22], suggest that ferroptosis is a different RCD pattern.

A small molecule high-throughput screen for RAS mutations identified ferroptosis. In 2003, erastin was recognized to be a selective agent that induces non-apoptotic cell death within tumor cell depending ST and RASG12V [23]. Additionally, the RAS/BRAF/MEK/MAPK pathway along with the voltage-dependent anion channel (VDAC) was implicated in this process, contributing to oxidative stress and mitochondrial dysfunction [24]. In the same way, RAS-selective lethal 3 (RSL3) was identified to promote a type of cell death regulated by iron in 2008 [25]. Next, the downregulation of GPX4 was shown to trigger non-apoptotic cell death and was inhibited by α-phenol and ALOX12/15 inhibitors [26].

2012, ferroptosis was introduced to characterize the specific mode of cell death. It is distinguished from other RCD based on its iron-dependence feature [11]. Erastin promotes ferroptosis through inhibiting the system xc^-^ to reduce the cystine uptake. Hepatostatin-1 was recognized as a highly effective suppressor of ferroptosis.

In 2014, GPX4 was identified as an important regulator of RSL3-induced and erastin-induced ferroptosis. RSL3 directly inactivates GPX4, lipid peroxidation, and results in ferroptosis [27]. In addition, knock out GPX4 leads to cell death. A spiroquinolamide derivative, listatproxin1, was reported to be effective in inhibiting ferroptosis [28]. In 2015, it was found that acyl-CoA inactivation can synthase long chain family member 4 (ACSL4) and lysophosphatidylcholine acyltransferase 3 (LPCAT3), inhibiting cells’ sensitivity towards ferroptosis. Meanwhile, the mutant p53 inhibits solute carrier family 7 member 11 (SLC7A11) express and reduces cystine intake, thereby enhancing the sensitivity towards ferroptosis [28]. In 2016, ALOXs were investigated to oxidize polyunsaturated fatty acyl (PUFA) via phosphorylase kinase G2-dependent iron pools, leading to ferroptosis. Whereas covalent inhibition of catalytic selenocysteine in GPX4 prevented PUFA hydroperoxide removal [29].

At the same time, it has been mentioned that Ferroptosis-Inducer-56 (FIN56) manages to promote GPX4 degradation and ubiquinone (CoQ10) consumption through mevalonate pathway, resulting in promoting ferrroptosis [30]. In 2017, ACSL4 was further recognized as a key part in ferroptosis by esterifying arachidonic acid (AA) and adrenic acid (AdA) into phosphatidylethanolamines (PEs). [31]. In 2018, studies highlighted the use of selenium in GPX4 to suppress ferroptosis [30].

Next, CD8^+^T cells’ ability in promoting tumor ferroptosis in immunotherapy was identified in 2019 [32]. Meanwhile, the yes-associated protein 1 (YAP) pathway was found to affect the levels of ACSL4 and transferrin receptor 1 (TFR1), which further regulates the sensitivity to ferroptosis [33]. In addition, the ferroptosis suppressor protein (FSP1), which was called apoptosis-inducing factor mitochondria-associated 2 (AIFM2) before, was found to be a new ferrroptosis resistant agent independent of the GPX4 system [34, 35].

In 2020, GTP cyclohydrolase-1 (GCH1) was discovered as ferroptosis inhibitor. It inhibits ferroptosis through 2 important mechanisms. First, GCH1 generates lipophilic antioxidant tetrahydrobiopterin (BH4), thereby inhibiting lipid peroxidation. Second, GCH1 upregulated levels of CoQ10 and inhibited ferroptosis [36, 37]. In addition, Zou et al. found that the key factor promoting the sensitivity and resistance of ferroptosis is oxidative organelle peroxisomes, which can synthesize polyunsaturated ether PLs, who are crucial for lipid peroxidation [38]. In year 2021, dihydroorotate dehydrogenase (DHODH) was found having the ability to reduce mitochondrial CoQ10 levels and is a suppressor of ferroptosis. Whereas DHODH inhibitors are able to promote ferroptosis, Mishima et al. suggest that they achieve this effect by inhibiting the FSP1 pathway [39].

In 2022, neutrophils, specifically polymorphonuclear (PMN) myeloid-derived suppressor cells (MDSCs), were found to be able to spontaneous ferroptosis. This finding aids in exploring the role of ferroptosis within the tumor immune microenvironment [40]. Notably, a whole-genome CRISPR activation screen in 2023 revealed that sex hormones can upregulate the expression of membrane-bound o-acyltransferase domains 1 and 2 (MBOAT1/2) which contribute in preventing the ferroptosis in cancer cells lacking GPX4 and FSP1 [41]. Overall, these studies enhance our understanding of ferroptosis mechanisms and regulation, highlighting its potential therapeutic implications.

## Drive mechanisms of ferroptosis

**Fig. 1:**
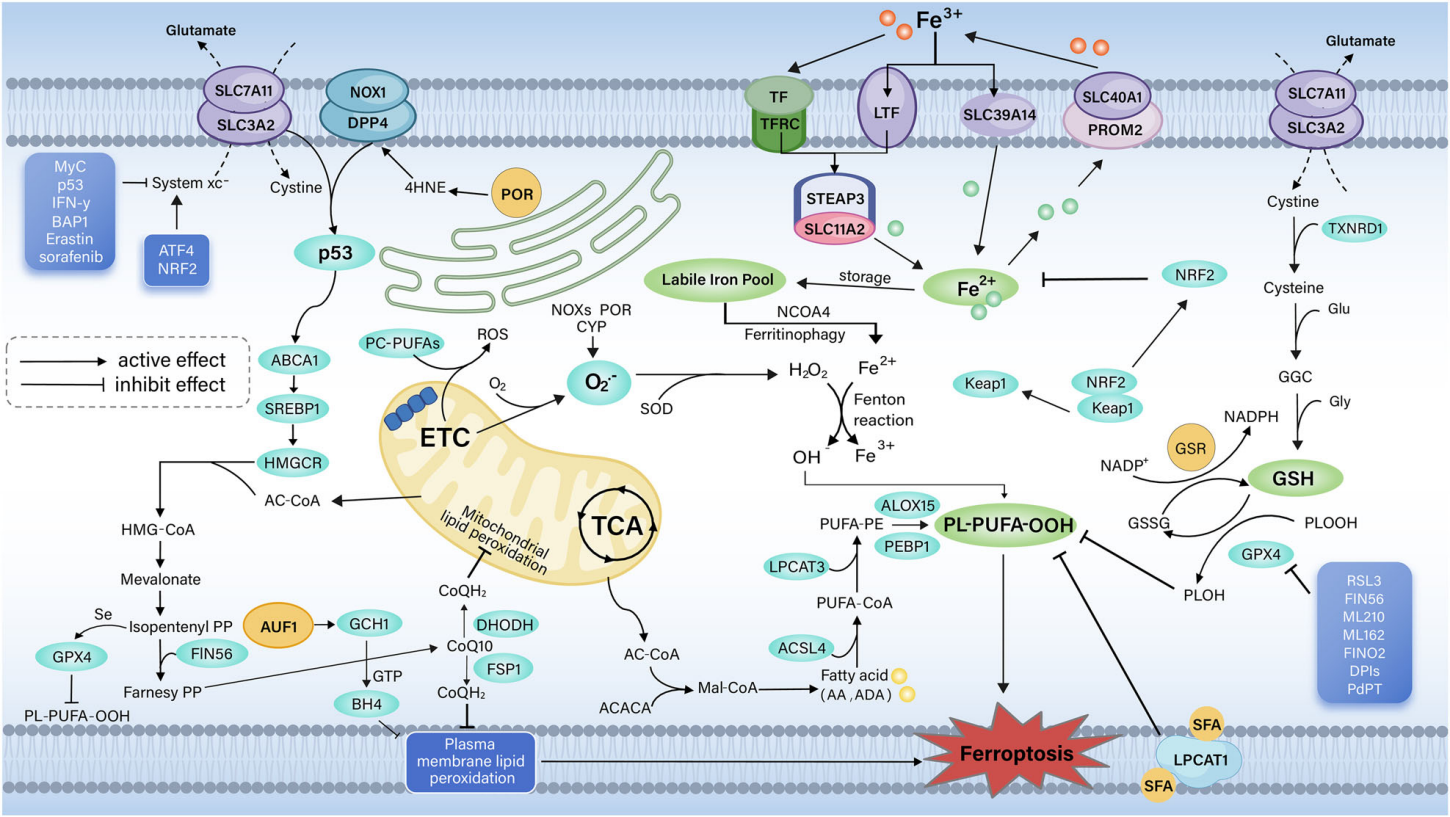
The mechanisms of ferroptosis. Ferroptosis is usually triggered by iron-dependent lipid peroxidation, its mechanisms are divided into driving mechanisms and defense mechanisms. Driving mechanisms are mainly through extrinsic or transporter-dependent pathways (e.g., system xc) and intrinsic or enzyme-regulated pathways, involving ROS production, enzymatic reaction, and metabolism of cysteine, glutamine, and iron. On the other hand, a variety of systems have evolved to antagonize ferroptosis in cells. GPX4 reduces lipid peroxides through GSH to avoid ferroptosis, while GPX4 is an important target for many ferroptosis inducers. The FSP1 system reduces CoQ10 and protects the integrity of the cell membrane; the DHODH system achieves this process on the mitochondrial membrane. When cells peroxidize, NRF2 regulates a variety of iron metabolism and GSH metabolism-related pathways to antagonize ferroptosis. 4HNE 4-Hydroxynonenal, AA arachidonic acid, ABCA1 ATP- binding cassette subfamily A member 1, Ac-coa Acetyl-coa, Mal malonyl, ACACA Acetyl-CoA Carboxylase Alpha, ACSL4 Long- chain fatty acid–CoA ligase 4, ADA adrenic acid, ALOX Arachidonate lipoxygenases, AUF1 AU-rich element-RNA binding factor, BH4 tetrahydrobiopterin, CoQ coenzyme Q, DHODH Dihydroorotate dehydrogenase, DPP4 dipeptidyl peptidase 4, ETC electron transport chain, FSP1 ferroptosis-suppressor-protein 1, GCH1 GTP cyclohydrolase 1, GGC γ-L-glutamyl-L-cysteine, GPX4 Glutathione Peroxidase 4, GSR glutathione disulfide reductase, GSSG glutathione disulfide, HMGCR HMG-CoA reductase, KEAP Kelch Like ECH Associated Protein, LPCAT lyso-phosphatidylcholine acyltransferase, NCOA4 nuclear receptor co-activator 4, NOX NADPH oxidase, NRF2 nuclear factor-erythroid 2-related factor 2, PC phosphatidylcholines, PE phosphatidylethanolamine, PEBP Phosphatidylethanolamine Binding Protein, PL phospholipids, POR P450 oxidoreductase, PUFA polyunsaturated fatty acyl, ROS reactive oxygen species, SFA saturated fatty acid, SLC3A2 solute carrier family 3 member 2, SLC7A11 solute carrier family 7 member 11, SOD Superoxide dismutase, SREBP sterol regulatory element binding protein, STEAP Six-Transmembrane Epithelial Antigen of Prostate, System xc cystine–glutamate antiporter, TCA cycle tricarboxylic acid cycle, TFRC transferrin receptor, TXNRD1 thioredoxin reductase 1.

Ferroptosis mechanisms are broadly separated to driving mechanisms and defense mechanisms (Fig. 1). The elevated accumulation of iron and excessive lipid peroxidation are main driving mechanisms, as discussed below.

### Iron metabolism

Iron is an indispensable micronutrient and has a significant impact on cell activity and cell death. In ferroptosis, iron accumulation can be directly involved in lipid peroxidation by Fenton reaction and can act as co-factor for enzymes. Therefore, it is crucial in regulating ferroptosis [42].

Iron homeostasis involves processes including iron uptake, utilization, storage, and export, which directly affect sensitivity to ferroptosis. Iron typically binds to transferrin (TF) in a Fe^3+^ form, thereby entering cells via transferrin receptor (TFRC) [43], then get reduced to Fe^2+^ by Six-Transmembrane Epithelial Antigen of Prostate (STEAP3) metal reductase in endosome. After that, they are released into the cytoplasm to form a labile iron pool (LIP) [44]. Excess divalent Fe^2+^ is excreted out of the cell through solute carrier family 40 member 1 (SLC40A1) and change into Fe^3+^ [45]. Any disruption in iron homeostasis can alter the LIP, thereby regulating ferroptosis happen.

The LIP induces ferroptosis mainly through several mechanisms: First, regulation of TFRC and solute carrier family 39 member 14 (SLC39A14) expression increases the uptake of exogenous iron. Examples include induction of ferroptosis in hepatocytes or Coxsackie B3 virus infected HeLa cells [46, 47]. In contrast, heat shock protein family B (small) member 1 (HSPB1, also known as HSP25 or HSP27) is able to remodel the cytoskeleton and prevent iron uptake, which in turn inhibits ferroptosis in cancer cells [48].

Secondly, Aconitase 1 increases Fe^2+^ intake and forms ferritin. Correspondingly, trilipoalanine ring finger protein maintains LIP capacity through breaking down mRNA transcripts and reduces the production of iron-binding proteins, especially iron-sulfur proteins [49, 50]. Thirdly, Nuclear receptor coactivator 4 (NCOA4)-dependent ferritin autophagy releases labile Fe^2+^ which promotes ferroptosis [51]. In contrast, glutamic-oxaloacetic transaminase 1 suppresses ferritin autophagy [52]. Last, the expression and inactivation of iron export agents can affect iron accumulation in cancer cell through transcriptional regulation and degradation [45, 53].

Additionally, organelles like Golgi apparatus and mitochondria also are crucial parts for LIP. Thus, regulation of iron homeostasis is discussed synthetically. Iron is also essential in many normal cellular activities, like DNA synthesis and oxygen transport. Therefore, to regulate the metabolism of iron without affecting these important roles and affect ferroptosis and then treat tumors, a comprehensive understanding of its molecular mechanisms is required.

### Lipid peroxidation

Lipid peroxidation is a biochemical process in which lipids in cell membranes and/or membrane organelles are attacked and oxidized by free radicals, such as ROS. This process generates lipid peroxides as well as other active complexes propagating and repeating spontaneously and oxidize nearby molecules. Increased or stimulated ROS up-regulate lipid peroxidation and induce RCDs. ROS are oxygen-containing active molecules resulting from the mitochondrial respiratory chain [54], Finton reaction [55], nicotinamide adenine dinucleotide phosphate oxidase (NOX) [56] as well as the reactions of enzyme [57, 58]. They are able to regulate the susceptibility to ferroptosis in cells and tissues [59].

#### Mitochondrial ROS

ROS are by-products generated by electron transport chain (ETC) within mitochondrial activities. It consists of multiple protein complexes embedded in the inner mitochondrial membrane. As cellular respiration, electron travels along the ETC, eventually reaches oxygen molecules. When electron flow coupling is imperfect or the membrane is compromised, electrons escape prematurely, especially from complexes I and III. These escaping electrons bind to molecular oxygen (O_2_) within mitochondria to form superoxide radicals (O_2_^−^). Superoxide dismutase (SOD) converts them to hydrogen peroxide (H_2_O_2_).

Mitochondrial ROS have been thought to have little to do with ferroptosis in the past. This idea primarily stemmed from the finding that cells lacking mitochondrial DNA, like P0 cells that are deficient in ETC catalytic subunits, continue to be susceptible to ferroptosis inducers [11]. However, nowadays research has shown that phospholipids containing diacyl polyunsaturated fatty acid tails may directly interact with ETC, thereby promoting the generation of ROS, then promotes lipid peroxidation as well as enhances ferroptosis sensitivity [60]. Similarly, H_2_O_2_ was once thought to be an apoptosis inducer, but new studies have found an induction of its ferroptosis [61]. However, the exact downstream pathways underlying these effects remain elusive. Identifying these downstream pathways is an important future research direction.

#### Fenton reaction

The Fenton reaction catalyzes H_2_O_2_ to become a hydroxyl radical (·OH) in conditions of transition metal ions, usually Fe^2+^. Hydroxyl radicals can activate several oxidative activities, such as lipid peroxidation and DNA damage. In lipids peroxides progress, Fe^2+^ induces the conversion of lipid hydroxyl radicals (LO·) and lipid peroxyl radicals (LOO·) from phospholipid hydroperoxides(LOOH) [62]. Therefore, affecting the sensitivity of ferroptosis depending on the absorption, use, storage, and export of iron by tissues [63].

#### NADPH oxidase

The NOX family facilitates electron changing from NADPH to O_2_ and generate O_2_^−^. They mainly include NOX1 to NOX5 and dual oxidase (DUOX) 1 and 2. The distribution, localization as well as functions of each member are different [64]. They take great parts in multiple signaling pathways, including those related to ferroptosis [65, 66]. For example, it has been shown that 4-hydroxynonenal (4HNE), a product of lipid peroxidation, can activate NOX1 and establish a positive feedback cycle that enhances cancer cell susceptibility to ferroptosis [56]. Therefore, it is necessary to find specific mechanism of NOX in activating ROS production and ultimately ferroptosis, and their therapeutic potential for related diseases.

#### Enzymatic reactions

Enzyme-dependent pathways are among the most prevalent and important in lipid peroxidation driven mechanisms. It contains multiple key enzymes, particularly ACSL4 and LPCAT3 [31, 67–69]. ACSL4 regulates PUFA-related acyl-CoA ester production through two pathways. One pathway combines PUFAs with CoA, generating PUFA-CoA, then converting to PE by LPCAT3 [70]. The other pathway involves sterol o-acyltransferase 1(SOAT1) in human pancreatic ductal adenocarcinoma (PDAC) cells and NM-6 leukemia cells deficient in solute carrier family 47 member 1 (SLC47A1) to produce fatty acid cholesteryl esters and enhance iron apoptosis sensitivity [71].

Some studies have reported related pathways, for example, LPCAT1 increases membrane PL saturation and decreases the level of membrane PUFAs via endogenous and exogenous saturated fatty acids (SFA) containing PLs [72]. As a result, this protects multiple tumor cells from peroxidation-induced membrane loss, which in turn inhibits ferroptosis [72]. In addition, acetyl-CoA carboxylase α (ACACA) may contribute in elongating of PUFAs, promoting FIN56-induced GPX4 protein degradation and induction of ferroptosis in HT-1080 cells [30].

The ALOX family includes multiple isoforms, such as ALOX5, ALOX12, and ALOX15 that promote the oxygenation of PUFAs. These enzymatic activities produce multiple bioactive lipid mediators. These mediators promote ferroptosis through variety of ways depending on the cell species [29, 73]. Phosphatidylethanolamine-binding protein 1 (PEBP1) can bind ALOX15 and upregulate lipid peroxide production [74]. Decreased levels of PEBP1 are related to cancer progression and are considered metastasis suppressors. Targeting ALOX15 has been shown to attenuate multimodal injury in mice. But this effect was not significant when GPX4 was depleted [28]. ALOX12 showed inhibition of TP53-mediated tumors in many cancer cell lines [75] and was not associated with ACSL4 [75].

Also independent from ACSL4-GPX4 pathway, pleckstrin homology domain family A member 2 (PHLDA2) in combination with ALOX12 mediates phosphatidic acid peroxidation and is able to inhibit tumors in normal mice [76]. In summary, each ALOX isomer possesses distinct and unique roles and mechanisms in ferroptosis.

Cytochrome P450 oxidoreductase (POR) promotes lipid peroxidation as well as ferroptosis [57, 58]. POR is highly expressed on the ER and can initiate PL peroxidation as well as ferroptosis apart from ALOX family by producing PL and 4HNE. Unlike the tissue-specific effects of ALOX, POR is widely expressed in various tissues. Therefore, targeting POR requires caution and precision [77]. In addition, prostaglandin E2 (PGE2) specifically activated ferroptosis in CD8^+^T cells, but not other RCDs [78, 79]. Furthermore, it limits IL-2-related antitumor activity [80]. An in-depth exploration of the role of the above pathways is essential for further development of diseases associated with the mechanism of ferroptosis and their targeted therapies.

#### NRF2 system

Nuclear factor erythroid 2-related factor2 (NRF2), a stress-induced transcription factor that is essential to maintain redox homeostasis. It has a conserved basic leucine zipper (bZIP) domain with an ability to form heterodimers with other proteins, which then bind to anti-oxidative stress elements (ARE) [81].

NRF2 often remains low levels and get regulated by Kelch-like ECH-related protein 1 (KEAP1). In oxygen-lacking conditions, they combine into complexes that degrade NRF2 by ubiquitination and reduce its free content. When cell peroxidation or other stress states are disrupted, this complex depolymerizes and releases NRF2 [82]. NRF2 levels are upregulated, enter the nucleus, bind to the original ARE, upregulate target genes’ transcription and expression, and then protect cells from peroxidation. NRF2 primarily controls target genes involved in intracellular redox reactions, including nearly all those associated with iron and GSH metabolism[83]. For example, it controls the functions of GPX4 system and the system xc^−^ as well as the metabolism of glucose [84].

Moreover, the p62-Keap1-NRF2 pathway is strongly associated with the elevated level of genes affecting ferroptosis and ROS metabolism [85]. Notably, NRF2 shows a bidirectional ability in regulating ferroptosis, whereby a decreased expression can stimulate the sensitivity of cancer cells to ferroptosis, while an increased level inhibits the ferroptosis process [86, 87]. Yau et al. reported that the activation of NRF2 upregulates the Metallothionein-1G, which in turn leads to decreased sensitivity to sorafenib and inhibits the lipid metabolism [88]. And in Acute Lung Injury (ALI), NRF2 functions through activating the production of antioxidant enzymes and genes, reducing the ferroptosis of alveolar epithelial cells and lung damage [89].

## Defense mechanism of ferroptosis

Ferroptosis occurs when oxidative and antioxidant systems are out of balance. Oxidative reactions occur intracellularly in which oxygen radicals are rich. Usually, cells do not peroxidize and damage death because efficient antioxidant systems continuously consume these free radicals. Ferroptosis often occurs due to failure of the antioxidant system. When antioxidant capacity is reduced, ROS generated by normal cellular activities can lead to peroxidation damage and then death even if the oxidative system is not active. Therefore, exploring the mechanisms and regulatory pathways of the antioxidant system is of utmost necessity. As research advances, the antioxidant system has progressively been categorized into two distinct types: those dependent on GPX4 and those independent of GPX4, which will be detailed below.

### GPX4-dependent pathways

#### GSH pathway

GSH ranks among the top plentiful and potent anti-oxidant agents. The imbalance of GSH metabolism often leads to the development of ferroptosis. GPX4 is one of the core proteins regulating ferroptosis in cells and relies on GSH to achieve antioxidant effects. GSH is an active substratum for GPX4, and when it is transferred to GPX4, GPX4 becomes oxidized glutathione (GSSG) and obtains antioxidant function. Upon activation, GPX4 catalyzes the conversion of PLOOH into PLOH, while also mitigating lipid peroxides and ROS within the membrane, thereby antagonizing ferroptosis. Conversely, when GSH levels are exhausted, GPX4 becomes inactive and nonfunctional, which subsequently results in lipid peroxidation and triggers ferroptosis [90].

GPX4 serves as a crucial target for numerous ferroptosis-inducing agents, with RSL3 being the most representative example [27]. Ferroptosis inducers RSL3 [27] and ML210 [91] irreversibly combined with the active selenocysteine site of GPX4 to promote ferroptosis. On the other hand, down-regulation of GPX4 protein expression was also able to promote ferroptosis. FIN56 [30] and PdPT [92], for example, are able to promote the breakdown of GPX4, thereby downregulating its intracellular expression, inducing ferroptosis as a result.

GPX4 fulfills a diverse array of functions, impacting ferroptosis as well as diverse cellular demises. Its multifunctionality is rooted in its participation in numerous pathways related to cellular homeostasis and stress responses.

#### System xc^-^

System xc^-^ was found to be a glutamate/cystine reverse transporter outside of the cell and consists of SLC7A11 and SLC3A2. It allows cystine and glutamate to enter and exit the cell in a 1:1 ratio. Intracellular cystine is rapidly reduced to cysteine by thioredoxin reductase 1 (TXNRD1).

Cysteine as a precursor for GSH synthesis functions as the bottleneck in the progress, which primarily consists of two steps. Initially, glutamate-cysteine ligase (GCL) facilitates the dipeptides (γ-glutamylcysteine) transform from glutamate and cysteine. Following this, glutathione synthase activates GSH synthesis from dipeptides and glycine [93]. Therefore, system xc^-^ emerged as one of the mechanisms regulating ferroptosis. Ferroptosis inducers Erastin [11], sorafenib [94] are able to suppress the expression of SLC7A11 to interfere with cystine uptake and thus affect GSH synthesis. Excessive extracellular glutamate concentration also impacts systemic xc^-^, preventing cystine entry, which in turn leads to GSH depletion triggering ferroptosis [95].

Moreover, 2-mercaptoethanol can reduce extracellular cystine to cysteine, which is then transported into the cell through the L system, thereby skipping the system xc^-^ and support GSH synthesis [96]. By this way, some health insurance can resist ferroptosis. For instance, Hayano indicated that the deletion of cysteine-tRNA synthase promoted the trans-sulfuration pathway and inhibited erastin-promoted ferroptosis [97].

### GPX4-independent pathways

#### FSP1-CoQ10 pathway

FSP1 is an ferroptosis regulator parallel to GSH. When the GSH-GPX4 antioxidant system was functioning properly, a decrease in FSP1 levels still triggered lipid peroxidation. Thus, the FSP1 antioxidant system begins in parallel with the GPX4 system. Mechanistically, FSP1 localizes to the cell membrane according to the structural features of mycolylation. It is responsible for reducing CoQ10 in membranes to reduced CoQ. CoQ10 is present in all biofilms and act as a mobile lipophilic electron carrier. The reduction was facilitated by FSP1 and NADPH. It plays the role of free radical trapping antioxidant (RTA) and subsequently interacts free radicals on cellular membrane, thereby inhibiting PL peroxidation. FSP1/CoQ10 system parallels GPX4 and system xc^-^ to maintain cellular redox balance [98].

#### DHODH-SOD2 pathway

GPX4 operates in both the cytoplasm and the mitochondria. In cytoplasm, GPX4 system works together with the CoQ10/FSP1 system to complement each other to maintain the overall antioxidant function of the cell. In mitochondria, the DHODH-SOD2 system take the place of CoQ10/FSP1 and cooperation with GPX4 system.

DHODH act as essential agents which functions within the de novo synthesis of pyrimidines in prokaryotes and eukaryotes, could reverse the increased sensitivity of SOD2 knockout cell to ferroptosis, it is also essential in cellular chromosome replication. DHODH is also related to the generation of ATP and ROS. It operates in a manner akin to the CoQ10/FSP1 system within cells, primarily helping to reduce CoQ10 to panthenol, which in turn provides antioxidants to clear the peroxides on the mitochondrial membranes, in turn maintaining membrane integrity. DHODH and GPX4 systems are complementary, and when the function of one system decreases, the other system increases, maintaining antioxidant capacity [99, 100].

#### GCH1-BH4 pathway

Two teams discovered another antioxidant mechanism parallel to the GPX4 system, the GCH1-BH4 pathway [36, 37]. GCH1 serves as one coenzyme for nitric oxide synthase as well as key enzyme required for BH4 synthesis [101]. GCH1 exhibits a strong antioxidant capacity. Overexpression of GCH1 can protect cells with GPX4 knockdown from ferroptosis, indicating that GCH1 functions through a pathway distinct from GPX4 [36].

Usage of BH2-BH4 to protect cells exposed to ferroptosis activators also demonstrates this idea [37]. AUF1 showed a positive correlation with GCH1 expression. Inhibit AUF1 was able to model increased apoptosis in cancer cells, whereas inhibition of GCH1 inhibited progression of ESCC cells [102]. Therefore, suppressing GCH1 might reduce BH4 levels, thereby inducing ferroptosis in EC cells. However, this hypothesis still requires validation, and the GCH1-BH2/BH4 pathway holds significant potential for further investigation.

In conclusion, multiple antioxidant systems act synergistically to regulate iron-induced oxidative stress and keep cellular redox homeostasis in humans. If one of these antioxidant systems is compromised or overloaded, other systems are connected to compensate and protect against oxidative damage and ferroptosis.

## Ferroptosis in the tumor microenvironment (TME)

**Fig. 2:**
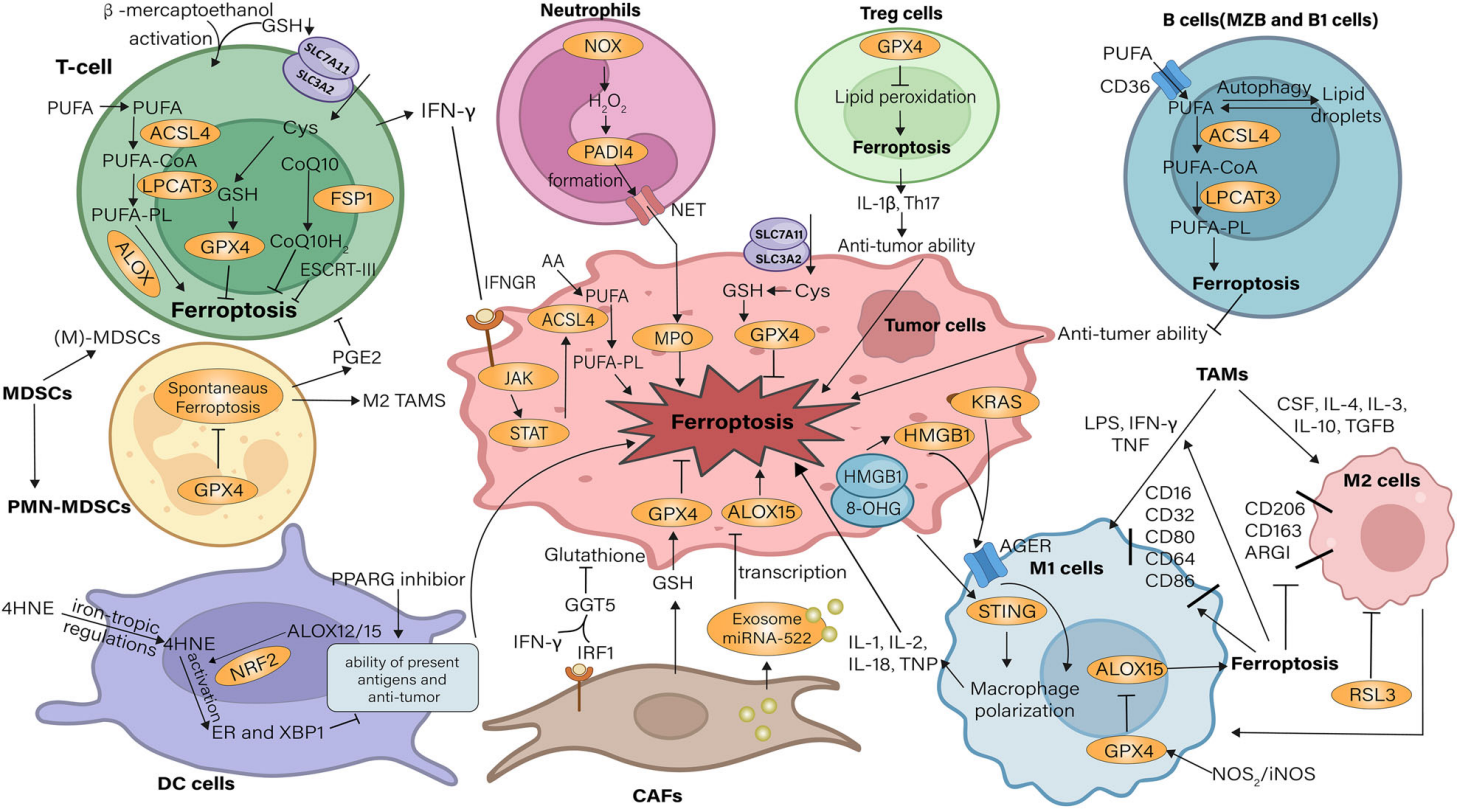
Effects of immune cells on ferroptosis in TME. T cells in TME release IFN-γ which down-regulate system xc^-^ to promote ferroptosis of cancer cells by GSH depletion. IFN-γ also promotes the differentiation of macrophages into M1 type, acquiring the ability to kill and phagocytoze cells, and inhibiting the development of tumors. DC is an important role in the occurrence of ferroptosis. Iron-tropic regulators further affect the activation of T cells by hindering the antigen presentation function of DC cells and hinder the occurrence of ferroptosis. CAFs are the main force of tumor resistance to ferroptosis, which can provide a large amount of GSH and Cys for tumors. However, IFN-γ secreted by T cells breaks down GSH by binding to CAF to generate GGT5, reducing tumor absorption. In the absence of GPX4, although the ferroptosis level of the tumor itself is increased, it also leads to the ferroptosis of Treg itself, leading to its loss of anti-tumor ability. On the other hand, it also leads to spontaneous ferroptosis of PMN-MDSCs and subsequent release of factors that inhibit the anti-tumor effects of T cells and TAMs. Thus, immune cells in the TME interact and work together to regulate ferroptosis. 8-OHG 8-hydroxyguanosine, CAF Cancer-Associated Fibroblasts, DC dentric cell, ER Endoplasmic reticulum, ESCRT endosomal sorting complexes required for transport, GGT γ-Glutamyl Transferase, HMGB1 High mobility group box 1 protein, JAK Janus Kinase, KRAS Kirsten rats arcomaviral oncogene homolog, MDSC Myeloid-derived suppressor cells, MPO myeloperoxidase, MZB Marginal Zone B And B1 Cell Specific Protein, NET Neutrophil Extracellular Traps, PADI4 Peptidyl Arginine Deiminase 4, PGE2 prostaglandin E2, PMN polymorphonuclear, PPARG peroxisome proliferator-activated receptor gamma, RSL3 RAS-selective lethal 3, STAT signal transducer and activator of transcription, STING stimulator of interferon genes, TAM tumor-associated macrophage, XBP1 X-box binding protein 1.

The TME is a dynamic complex ecosystem that includes cancer cells, stromal cells, different immune cell subsets, the blood and lymphovascular system, and various non-cellular components [103]. In TME, bidirectional communication between cancer cells and the microenvironment regulates tumor growth [104]. In particular, dying tumor cell interact with immune cell via a variety of signals during ferroptosis, in turn modulating immune responses (Fig. 2). On the contrary, immune cell also have the ability to regulate cancer cells' susceptibility to ferroptosis by releasing mediators. Pharmacological screening, for example, revealed that T cell has higher sensitivity towards FINs rather than tumor cell. Iron-promoting stimulation causes ferroptosis in cancer cell as well as tumor-infiltrating immune cell [105]. Accordingly, immune cell ferroptosis impacts its immunomodulatory function and ultimately manifests itself in TME as reprogramming tumor progression. Therefore, ferroptosis plays various complex roles in TME, especially between immune cells and tumor cells are discussed below.

### Ferroptosis in T cells

Multiple studies conducted both in vitro and in vivo have demonstrated that the activities and functions of CD8^+^T and CD4^+^T cells are significantly associated with lipid peroxidation and ferroptosis. For example, SLC7A11 is virtually absent from human naive CD4^+^T cells. However, upon T cell activation, its expression increases substantially [106, 107]. To activate and proliferate, T cells need a reductive extracellular environment to preserve their intracellular GSH levels. This can be accomplished by supplementing the culture medium with β-mercaptoethanol during in vitro experiments [108].

When GPX4 is depleted or supplemented with GPX4 pathway ferroptosis inducers (e.g., RSL3, ML210), lipid peroxidation happens within T cells induced in vitro, which is accompanied by ferroptosis. In contrast, CD8^+^T cells were protected from ferroptosis when GPX4 and FSP1 were overexpressed, or ACSL4 was knocked down [105, 109]. One study showed that GPX4-deficient CD4^+^T or CD8^+^T are unable to exercise immune function during acute infection. This deficiency can be corrected by adding vitamin E, a powerful fat-soluble antioxidant.

CD8^+^T cells activation can increase the susceptibility of neighboring non-T cell towards ferroptosis, especially when in tumor therapy. Mechanistically, CD8^+^T cell releases IFN-γ, a cytokine reducing systemic xc^-^ level, thereby reducing cystine utilization by cancer cells, which in turn allows GSH depletion to trigger ferroptosis [32]. Taken together, it is believed that iron-tropic cancer cells are immunogenic and capable of activating CD8^+^T cells to mediate antitumor immune responses [110]. Therefore, it is suggested that CD8^+^T cell can initiate immunogenic cell death by inducing ferroptosis, and can amplify anti-cancer immune responses as well as broaden ideas for cancer therapy.

Activated Treg cells are important means of antitumor immunity and autoimmunity [111]. In the absence of GPX4, activated Tregs have higher sensitivity to ferroptosis thereby promoting the generation of pro-inflammatory agent IL-1β, which in turn promotes T helper 17 (Th17) functions. This mechanism undermines the immunosuppressive role of Tregs in TME, thereby enhancing cancer cell development. In experiments with Treg specific deletion GPX4 mice, the addition of ferroptosis inhibitors was able to restore tumor burden [112]. Thus, inhibition of GPX4-targeted ferroptosis in tumor Tregs could be a direction to reprogram TME and treat cancer. However, on the other hand, the non-selective absent of GPX4 in Tregs leads to deleterious autoimmunity. Therefore, in future studies, attention should be paid to determine how Tregs that do not affect healthy tissues and selectively target tumor-infiltrating Tregs when inducing ferroptosis.

### Ferroptosis in B cells

B cells are markedly heterogeneous and important in TME. So far, the involvement of ferroptosis in the homeostasis and immune response of tumor-infiltrating B cells remains underexplored. Mature B cells are broadly divided into follicular or B2 cells, marginal zone B cells (MZB) and B1 cells.

New research suggests that MZB cells and B1 cells are sensitive to GPX4 inhibitors. They found that GPX4 is essential in MZB cells and B1 cells during development, maintenance. Moreover, B1 and MZB cells lacking GPX4 accumulate lipid peroxidation and ferroptosis due to their high uptake of fatty acids (including OX-LDL) through CD36 to sustain metabolic functions [113]. In detail, fatty acids act as raw materials in PL-PUFA-OOH synthesized through the ACSL4-ALOX pathway, and its accumulation initiates ferroptosis, in addition, OX-LDL stimulation increased ACSL4 expression which activates ferroptosis while decreased GPX4 and GCH1 expression which inhibits ferroptosis [114]. This observation broadens the insights into the mechanisms underlying B cell redox homeostasis [113].

Two more studies further emphasize the importance of ferroptosis in maintaining B cell homeostasis. Firstly, B1 cells depend more on exogenous fatty acid uptake as well as autophagy mobilization for survival and metabolism than B2 cells [115]. Fatty acids exist as lipid droplets, which can inhibit PUFA oxidation [116]. Its autophagy is able to upregulate intracellular fatty acid levels, which in turn promote siderophil cell death [117]. Thus, lipid droplet autophagy may regulate B cell function through ferroptosis. Second, lipid peroxidation induced by erastin can down-regulate the expression levels of bone morphogenetic protein family members [118], thereby induce the proliferation and differentiation of B cell and NK cell [119].

### Ferroptosis in dendritic cells (DCs)

DCs are considered potent initiators in presenting antigens and activating the responses of T cell, they have the capacity to eliminate cancer cells within tumor TME [120]. Recent research has highlighted that iron-regulating factors can compromise the anti-tumor functions of tumor-infiltrating DCs. In ovarian cancer-associated DCs, damaging molecules such as ROS and lipid peroxidation by-products tend to accumulate [121, 122]. The buildup of these molecules promotes the activation of the ER stress response as well as the X-BOX-binding protein 1 pathway. These activations, thereby compromise DCs’ capacity to present antigens and trigger antitumor immune reactions [123].

It is worth mentioning that GPX4 inhibitors, can trigger ferroptosis in DCs in a peroxisome proliferator-activated receptor (PPAR) dependent condition. PPAR gamma (PPARG) gene inhibition can significantly restore the antitumor functions of damaged iron-tropic DCs in vivo [124]. In addition, ALOX12/15 is able to disrupt DC maturation and activation through the NRF2 pathway in inflammatory models, but the mechanism by which it elicits antitumor immune dysfunction in DCs remains uncertain in TME [125]. Overall, ferroptosis was able to occur in DCs and attenuate their antitumor function.

### Ferroptosis in tumor-associated macrophages (TAM)

TAM can differentiate into immunostimulatory M1 or immunosuppressive M2 [126]. M1 types are characterized by the expression of CD16, CD32, CD80, CD86, CD64, and nitric oxide synthase 2 (NOS2/iNOS) as their defining markers, whereas M2 macrophages predominantly expressed CD163 and CD206. LPS, IFN-γ, and TNF drive macrophages to adopt the M1 phenotype, which in turn stimulates the release of agents like IL-1, IL-12, IL-18 and TNF [127]. On the other hand, the activation of M2 type are triggered by CSF1, IL-4, IL-13, IL-10, and TGFB1 [127].

Emerging studies have gradually shown that macrophage polarization and ferroptosis may interact mutually at cellular level or act in a downstream dependent manner above other cells. It is worth mentioning that while GPX4, ACSL4 and LPCAT3 showed little difference in expression levels between M1 and M2, M1 showed a stronger ability to recover from ferroptosis [128]. This ability is attributed to the increased levels of iNOS and NO^•^ within it. They can replace GPX4 thereby suppress ALOX15-induced lipid peroxidation as well as ferroptosis. Ferroptosis inducer RSL3 induced ferroptosis within M2 cells but did not affect M1 cells [128].

In addition, during ferroptosis, stimulation of ferroptosis reprograms TAM into an anti-tumor M1 phenotype, which in turn suppresses tumor growth. For example, APOC1 or SLC7A11 can promote ferroptosis in TAM, as shown by increased iron content, down-regulation of iron-resistant ferroptosis regulators (GPX4, NRF2, SLC7A11, and GSH), and associated significant mitochondrial changes [129, 130]. This type of ferroptosis promotes CD86 expression in the M1 phenotype, downregulates CD206, CD163, and ARG1 expression in the M2 phenotype, and ultimately inhibits pretumor M2 polarization and HCC development. In addition, the M2 phenotype can also be transformed into the M1 phenotype.

FINs-loaded iron funds belong to organic frameworks (RSL3 or dihydroartemisinin) that are capable of doing so as ferroptosis nanoparticles. From the results, M2 to M1 transition provoked antitumor activity of TAM, acquired phagocytic cells, killed cells and metastasis inhibition [131–133]. In summary, targeting ferroptosis in TAM, eliminating M2 or reprogramming it into tumoricidal M1 cells thereby delaying cancer progression, is a feasible therapeutic direction.

### Ferroptosis in natural killer cells

NK cells are a subset of natural cytotoxic lymphocytes, have been thought to avoid surveillance by CD8^+^T cells and eliminate malignant cells [134]. In TME, a special class of NK cell, tumor-associated NK (TANK) cell, plays crucial role. However, due to oxidative stress caused by lipid peroxidation, glycolysis, one of the key metabolic ways of TANK cell effector function, is reduced, which in turn inhibits its anti-tumor function and makes cancer progression.

In the gastric cancer TME, L-Kynurenine (L-KYN), which is a metabolite derived from tryptophan, was documented to provoke lipid peroxidation thereby induce ferroptosis in NK cells [135]. However, overexpression of GPX4 could render NK cells resistant to such ferroptosis and enhance their antitumor effects. Similarly, NRF2 was able to coordinate metabolism in TANK cells, restoring glucose metabolism and oxidative phosphorylation (OXPHOS) under glutamine (Gln) replete conditions [136]. The above studies further elucidate the relationship between NK cells and ferroptosis. However, this relationship is currently not clear and remains to be explored in depth.

### Ferroptosis in MDSCs

MDSCs are pathologically activated immature cells with strong immunosuppressive effects and heterogeneity [137]. There are broadly two subgroups: PMN-MDSCs and monocytes (M)-MDSCs [138]. Among them, tumor-infiltrating PMN-MDSCs are sensitive to ferroptosis, even are able to spontaneous ferroptosis in the TME. Downregulation of GPX4 exacerbates this process. This type of ferroptosis leads to a decrease of PMN-MDSCs, but the release of PGE2 and lipid peroxide increases [40]. They can limit T cell’s effect in anti-tumor, support the inhibitory function of TAM, ultimately leading to tumor growth [139]. In summary, ferroptosis induction can inhibit MDSC viability, further studies must comprehensively consider the complex regulatory mechanisms of MDSC in TME.

### Ferroptosis in cancer associated fibroblasts (CAFs)

CAFs are key promoters for tumor progression in TME. Recent researches have revealed their important role in ferroptosis balance. CAFs are able to enhance cancer cell resistance to ferroptosis, and one of the mechanisms is to provide critical antioxidants, especially GSH and Cys [140]. Another study proposed that CAFs release exosome-derived miR-522, which post-transcriptionally is able to inhibit the translation of ALOX15 mRNA in cancer cells. This disrupted the conversion of PUFA-PE to PL-PUFA-OOH resulting from ALOX15 binding to PEBP1, which in turn protects cells from ferroptosis [141].

However, it was not without loopholes. CD8^+^T cells were found to counteract this effect. They release IFN-γ, which attaches to the interferon regulatory factor 1 (IRF1) receptor on CAFs and stimulates the transcription of γ-glutamyltransferase (GGT5). GGT5 is able to degrade extracellular GSH, making the antioxidant potential of CAFs decreased. In addition, IFN-γ was able to activate the JAK/STAT pathway of CAFs to repress systemic xc^-^ transcription. This inhibition eventually leads to decreased intracellular GSH and Cys levels, and cancer cells are susceptible to ferroptosis [142].

## Metabolism reprogramming and ferroptosis in TME

**Fig. 3:**
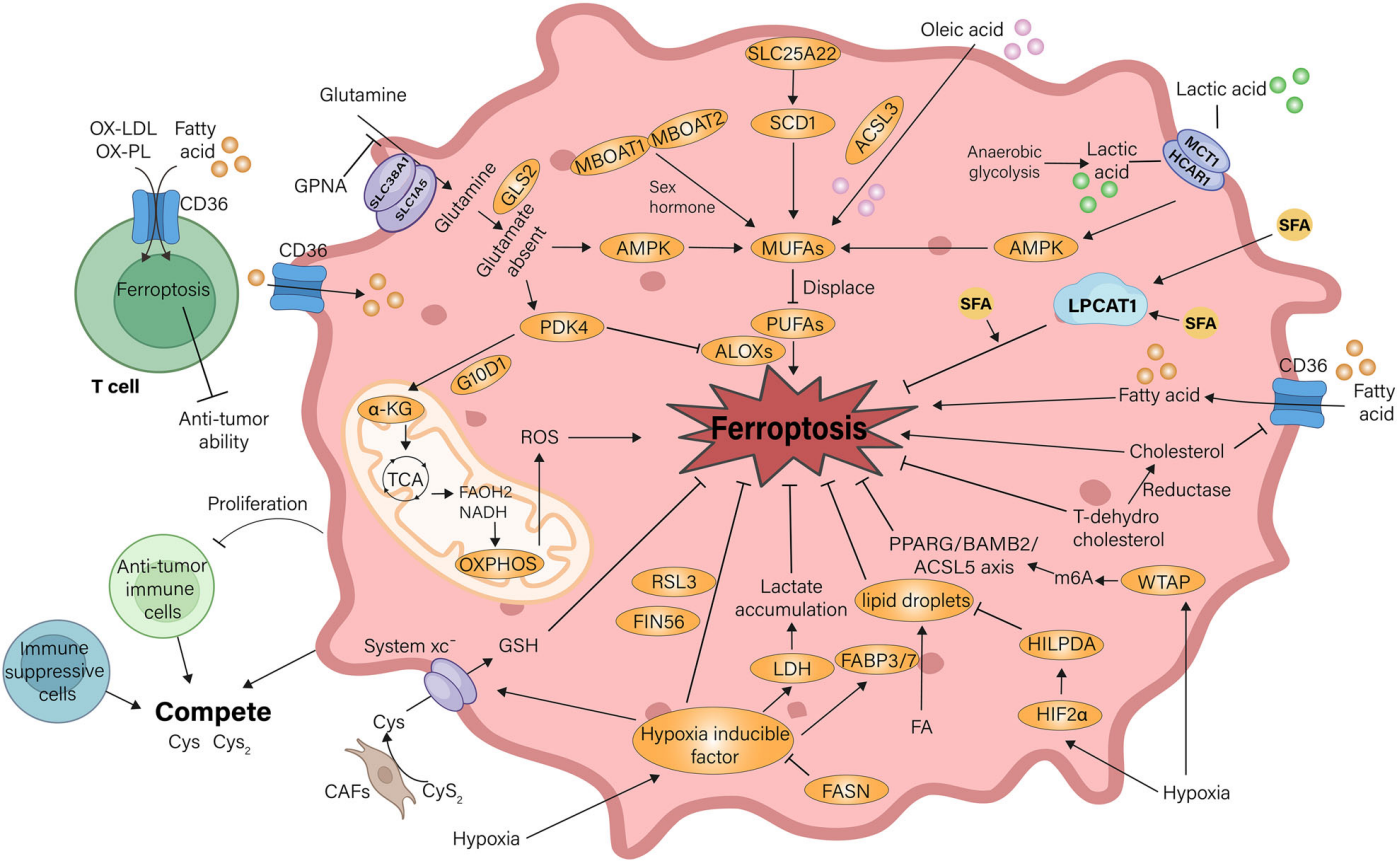
Metabolism reprogramming and ferroptosis in TME. Ferroptosis is closely related to REDOX metabolism and environmental regulation in the tumor microenvironment, including lipid metabolism, glucose metabolism, amino acid metabolism, acidification stress, hypoxia. Glucose provides the main energy for cell proliferation through aerobic glycolysis. When glucose is absent, ALOX mediates the overexpression of pyruvate dehydrogenase kinase 4 (PDK4), which eventually leads to the occurrence of lipid peroxidation. Mitochondrial glutamate transporter carrier family 25 member 22 (SLC25A22), which regulates GSH expression, can antagonize ferroptosis by promoting stearyl-CoA desaturation enzyme (SCD1) expression to produce MUFA. PUFA, MUFA, and SFA compete in regulating ferroptosis sensitivity. Fatty acids can up-regulate the expression of CD36 in CD8 + T cells and promote the uptake of OX-LDL and OX-PL. This function triggers T-cell ferroptosis, which affects antitumor immunity. In cancer cells, cholesterol antagonizes this process. Cancer cells utilize high levels of anaerobic glycolysis, resulting in the production and release of large amounts of lactate, which leads to extracellular acidification, and elevated lactate levels simultaneously stimulate the AMPK pathway, resulting in increased cell membrane MUFA and antagonize ferroptosis. Hypoxia-regulated transcription of HIF1α up-regulates fatty acid binding proteins 3 and 7 (FABP3/7), which promotes the production of lipid droplets and thus prevents ferroptosis. At the same time, hypoxia promotes WTAP mediated m6A regulation of PPARGC1A/BAMBI/ACSL5 axis and reduces ROS production, thereby down-regulating lipid peroxidation level and ferroptosis. However, HIF 2α promotes the conversion of lipid droplets to fatty acids, leading to the accumulation of PUFA and the occurrence of lipid peroxidation. α-KG α-Ketoglutaric acid, AMPK Adenosine 5‘-monophosphate (AMP)-activated protein kinase, CAF Cancer-Associated Fibroblasts, FABP fatty acid-binding protein, FASN Fatty Acid Synthase, FIN56 Ferroptosis-Inducer-56, GLS2 Glutaminase, GPNA L-γ-Glutamyl-p-nitroanilide hydrochloride, HCAR1 Hydroxycarboxylic Acid Receptor 1, HIF hypoxia-inducible factor, HILPDA Hypoxia Inducible Lipid Droplet Associated, LDH lactate dehydrogenase, LPCAT lyso-phosphatidylcholine acyltransferase, MBOAT membrane bound O-acyl transferase, MCT1 Monocarboxylate transporter 1, MUFA mono-unsaturated fatty acids, OXPHOS oxidative phosphorylation, PDK3 Pyruvate Dehydrogenase Kinase 3, PPARG Peroxisome proliferator-activated receptor gamma, SCD Stearoyl-CoA Desaturase, WTAP Wilms’tumor 1-associating protein.

Ferroptosis is inextricably linked to redox metabolism and environmental regulation of cell. Within TME, cells adapt their metabolic profiles as well as intensities in response to environmental stress. These adaptations, along with reprogrammed metabolism, are key characteristics of cancer. Nutrient competition in the TME, coupled with conditions like acidosis and hypoxia, can trigger ferroptosis, which in turn contributes to cancer progression (Fig. 3).

### Glucose metabolism and ferroptosis

In TME, glucose provides the main energy for cell proliferation through aerobic glycolysis, especially for fast proliferating cells, including tumor cell and activated T cells [143]. Cancer cell as well as bone marrow cell can consume the majority of glucose within TME [144]. Additionally, glucose consumption appears to antagonize ferroptosis in cancer cells and immunosuppressive cells, while reducing the anti-tumor function of activated T cells [145].

A related study hypothesized that under glucose-depleted conditions, tumor cell would survive by storing ATP, thereby avoiding ferroptosis. Energy deficiency can prompt the triggering of AMP-activated protein kinase (AMPK), which subsequently inhibits PUFA generation thereby counteracts ferroptosis [146]. Another study showed that increasing glucose intake through glucose transporter 1 enhanced PDAC to ferroptosis.

Glucose elevates the levels of glycolysis, which in turn promotes pyruvate oxidation, the tricarboxylic acid (TCA) cycle, and fatty acid synthesis. When glucose is absent, ALOX5 mediates pyruvate dehydrogenase kinase 4 (PDK4) overexpression, and the TCA is inhibited, ultimately leading to the development of lipid peroxidation [147]. Significantly, the antitumor effect of CD8 + T cells was reduced when grapevine he was absent [148]. In contrast, Tregs are not subject to this restriction. It preferentially uses lactate metabolism as an energy source, thereby maintaining its inhibitory activity.

Although glucose deprivation appears to have a tumor-promoting effect, the metabolic dangers of its exposure also need to be considered [149]. New research suggests that tumor cell that has over-expressed systemic xc^-^ level are susceptible to conditions of low glucose expression in TME. Targeted suppresses system xc^-^ expression, in contrast, provokes ferroptosis [150].

### Amino acid and ferroptosis

Proliferation of cancer cells and tumor-induced vascular abnormalities result in low levels of Cys2, Cys, and Gln in the TME [151, 152]. This provokes intensive oxidative stress, rendering cells more dependent on the GSH-mediated antioxidant system [153, 154]. Research has demonstrated that antitumor immuno-cells engage in competition with cancer cell as well as immuno-suppressive cell for cysteine (Cys) and cystine (Cys2) [155].

A study on PDAC and CLL showed that cancer cells evade this ferroptosis through some pathways. Examples include the effects of systemic xc^-^ knockout PDAC cells and CAFs [156, 157]. Specifically, CAFs are able to metabolize Cys2 to Cys and transport it into the TME with native cancer cells. Here, systemic xc^-^ knockout PDAC cell intakes them through alternative transporters such as ASCT1, ASCT2, LAT1 and SNATs [157]. In addition, bone marrow stromal cells characterized by increased level of system xc^-^ were found to secrete Cys into cells with decreased system xc^-^ expression and increased demand for GSH in the surrounding system [158, 159].

Gln serves as a vital energy provider of both immune and tumor cells. Many researches revealed that most tumors preferentially utilize exogenous Gln and emphasize its indispensable metabolic role. Gln acts as a supplementary fuel for the TCA cycle. It enters in cells primarily throuogh SNAT1 and ASCT2 and is subsequently metabolized by glutaminase (GLS) to Glu. It is subsequently hydrolyzed to α-ketoglutarate (α-KG), which is a key step in the TCA cycle [160–162]. Gao explored what mechanisms drive glutaminolytic enzymes in the regulation of CDI ferroptosis [163, 164]. Specifically, Glu exchanges to Cys via the system xc^-^, which in turn promotes CDI ferroptosis. Subsequent studies showed that extraneous Gln is essential for exchanging Cys exchange in the system xc^-^, accounting for approximately half of all Gln [165].

### Lipids and cholesterol in ferroptosis

Lipids are a class of molecules distinguished via their diverse structural characteristics. Some lipids can inhibit the effects of PUFA lipid peroxidation by competing or acting directly as antioxidants. For instance, ACSL3-dependent production of MUFAs can directly displace PUFAs from membrane PL and inhibit lipid peroxidation and ferroptosis [166].

Mitochondrial glutamate transporter solute carrier family 25 member 22 (SLC25A22), which regulates GSH expression, can also antagonize ferroptosis by promoting MUFA production from stearoyl-CoA desaturase (SCD1) expression [167]. Similarly, exogenous oleic acid reduced PUFA expression and attenuated acute iron overload injury in mice model. However, given that iron overload–related injuries generally progress slowly in humans, future researches are expected to demonstrate the protective effect of oleic acid [168].

Another source of MUFA generation is associated with MBOAT1 and MBOAT2 and is regulated by sex hormone receptors. These enzymes can reorganize the cellular phospholipid profile, which in turn produces phospholipids containing MUFAs, thereby preventing ferroptosis [41]. It has also been shown that SFA dependent on LPCAT1 antagonizes ferroptosis. Taken together, PUFA, MUFA, and SFA compete in regulating ferroptosis sensitivity.

Cyclin-dependent kinase inhibitor 2 A (CDKN2A) is a protein functions in inhibiting cancer, which also controls the G1-S phase transition in cell cycle, which thereby regulates cell growth [169]. When it is absent, lipid metabolism is subsequently altered, and ferroptosis sensitivity in glioblastoma is also enhanced. Similarly, inhibition of TP53 and CDK4/6 function promoted the arrest of cell cycle and improved the susceptibility to GPX4 inhibitor-regulated ferroptosis in some models [170]. In contrast, cell cycle arrest in human RCC cell lines inhibited ferroptosis by elevating lipid droplet content [171]. Combined, the sensitivity to ferroptosis varied between cell cycles.

Some endogenous lipid metabolites limit lipid peroxidation and thus ferroptosis and tumor progression. For example, 7-dehydrocholesterol, the precursor of cholesterol, contains structural properties brought about by the 5,7-unsaturated double bond that prevent phospholipid peroxidation and inhibit ferroptosis. In contrast, its reductase is able to promote ferroptosis by converting it to cholesterol. Therefore, regulation of 7-dehydrocholesterol levels provides novel targets for the treatment of ferroptosis-related cancers [172–175].

In addition, cholesterol in TME can regulate CD36 expression up-regulation in mice melanoma B16 and mice multiple myeloma infiltrating CD8^+^T cells and activate the uptake of fatty acids like AA. This function leads to CD8^+^T cell undergo lipid peroxidation and ferroptosis, thereby dysfunction, which in turn impacts the anti-tumor immunity in mice [176]. Clinical evidence shows a decreased levels of CD36 expression in long-term surviving melanoma and multiple myeloma patients, validating this process.

Further researches have revealed elevated concentrations of several classes of lipids in the TME and accumulation of these in CD8+ tumor infiltrating lymphocytes (TILs). These lipids accumulation was associated with increased expression of CD36, on CD8+ TILs, which also correlated with progressive T cell dysfunction. Cd36^−/−^ T cells retained effector functions in the TME, as compared to WT counterparts [177].

Mechanistically, CD36 promoted uptake of OX-LDL into tumor infiltrating T cells and this induced lipid peroxidation as well as downstream activation of p38 mitogenic activation of protein kinase (MAPK). Also, reduce the OX-LDL intake in tumor cells, thereby protect the tumor cell from ferroptosis. To conquer this, the inhibition of p38 restored effector T cell functions in vitro, and overexpression of GPX4 restored functionalities in CD8+ TILs in vivo [177].

Thus, an oxidized lipid-CD36 axis promotes intra-tumoral CD8^+^T cell dysfunction and serves as a therapeutic avenue for immunotherapies. Nevertheless, they failed to explain how CD36 selectively takes up lipids to achieve these effects [177].

### Acidification stress and ferroptosis

Tumor cell utilizes anaerobic glycolysis at high levels, resulting in the generation and emission of large numbers of lactic acid, which thereby leads to acidification outside the cell [178]. Cancer cells are able to take up them via transporters such as hydroxycarboxylate receptor 1 (HCAR1) and monocarboxylate transporter 1 (MCT1). The overexpression of lactate simultaneously stimulated the AMPK pathway, resulting in increased cell membrane MUFA and decreased ACSL4, antagonizing ferroptosis [179]. Therefore, acidic environment can promote tumor progression via antagonizing ferroptosis.

Within many tumors, lymphatic-metastasis occurs earlier [180, 181]. Cancer cells in lymph exhibit greater resistance to ferroptosis [182, 183]. One study proposed that in blood, high oxidative stress provoked ferroptosis within tumor cells, while this effect in lymph was far lower than in blood [184, 185]. It is worth mentioning that lymph contains large amounts of oleic acid, a MUFA. In response to fatty acid transporters, tumor cells can reduce the proportion of PUFAs. Cancer cells were safeguarded from ferroptosis in an ACSL3-dependent way by oleic acid. Additionally, the ratio of GSH/GSSG in lymph is substantially higher than that in blood, and it is also able to reduce oxidative stress and then reduce ferroptosis.

### Hypoxia and ferroptosis

Hypoxia was shown to be a normal feature of tumors and occurs within around 90% of tumors, promoting tumor progression and antagonism to treatment [186–188]. Hypoxic responses are primarily controlled by hypoxia-inducible factors (HIF), which are generally highly expressed within cancer cells, rendering cancer cells adapt to hypoxic environments [189–191].

HIF has a dual function in controlling ferroptosis and cancer therapy. In lung cancer cells, hypoxia can induce HIFα expression, which subsequently limits ferroptosis by RSL3/FIN56. Mechanistically, hypoxic HIF1α is transcribed to upregulate fatty acid binding proteins 3 and 7, in turn enhancing fatty acid uptake and lipid storage [192]. This process promotes the generation of lipid droplets, thereby circumventing ferroptosis. On the other hand, hypoxia can promote HIF1α-mediated transcription of lactate dehydrogenase (LDH) and SLC7A11, activate intracellular lactate accumulation and cystine uptake, and promote tumor resistance to ferroptosis through the lactate/GPX4 pathway [193].

In addition, hypoxia causes Wilms’tumor 1-associating protein (WTAP)-mediated m6A to regulate PPARG/BAMB2/ACSL5 axis, reduce ROS generation, and then down-regulate lipid peroxidation levels and ferroptosis [194]. Fatty acid synthase is significantly highly expressed in highly resistant cancers. It is able to bind HIF1α, down-regulate its ubiquitination and degradation, promoting its nuclear ectopy. This can lead to cancer cells becoming resistant to ferroptosis and some drugs [195]. Inhibition of hypoxia-activated HIF1α signaling may therefore be able to reverse resistance by promoting ferroptosis [196].

Remarkably, hypoxia is expressed in a HIF2α dependent manner as well by up-regulating lipid and iron-regulated genes. Activation of HIF2α can lead to increased lipid droplet transfer to fatty acid, which in turn drives PUFA accumulation and lipid peroxidation. This helps to promote the sensitivity of tumor cells of ferroptosis [197]. Above studies revealed the convoluted system of hypoxia regulating ferroptosis and provide possible important ways to target hypoxia signaling to treat cancer.

## Targeting ferroptosis to treat EC

### Targeting GPX4 in EC

**Fig. 4:**
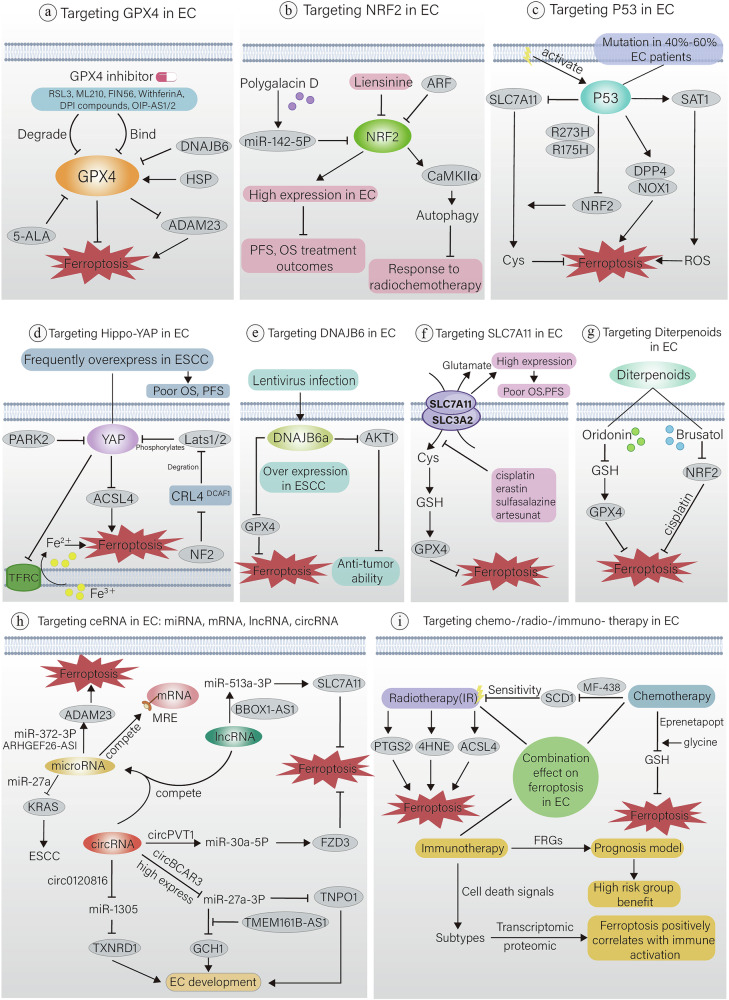
Targeting ferroptosis to treat EC. **a** GPX4 inhibitors inhibits GPX4 functions mainly through binding to its active site or degrading it and induce ferroptosis. **b** NRF2 regulates EC ferroptosis in many aspects, high levels of NRF2 are often associated with poor prognosis. polygalacin D, liensinine and ARF can inhibit the expression of NRF2. **c** radiotherapy induces the activation of P53, suppresses the expression of SLC7A11, leading to EC cell ferroptosis. **d** YAP mediates the expression of transferrin receptor 1 (TFRC) and ACSL4, in turns regulates sensitivity to ferroptosis, high levels of YAP often indicate poor OS and PFS. **e** DNAJB6 induce ferroptosis through down-regulating GPX4. **f** several compounds have been identified as inhibitors of SLC7A11 and shown significant treatment effect, including the ferroptosis-inducing agent erastin, sulfasalazine, cisplatin, sorafenib, and artesunate. **g** oridonin and brusatol, two kinds of diterpenoid, can induce EC cell ferroptosis by GPX4 and NRF2 pathway. **h** ceRNA is a hotspot in EC ferroptosis researches, providing various target for treatment. **i** radiotherapy, and chemotherapy showed great effect in EC ferroptosis, it is more significant when combined. In recent years, breakthrough progress has been made in the application of immunotherapy in patients with EC. Prognosis model has shown that ferroptosis positively correlates with immune activation. The combination of radiotherapy, chemotherapy and immunotherapy has great therapeutic potential in EC ferroptosis. 5-ALA 5-Aminolevulinic acid, ADAM23 A Disintegrin And Metalloproteinase Domain 23, AKT1 Threonine Kinase 1, ARF Auxin response factors, ARHGEF26 Rho Guanine Nucleotide Exchange Factor 26, BBOX-AS1 Gamma-Butyrobetaine Hydroxylase 1-Antisense RNA 1, CAMK Calcium–calmodulin dependent protein kinase, CRL Cullin-RING ligases, DNAJB6 DnaJ Heat Shock Protein Family (Hsp40) Member B6, FZD3 Frizzled Class Receptor 3, HSP Heat shock protein, MRE Meiotic Recombination, PARK Parkinson disease protein, PTGS2 prostaglandin-endoperoxide synthase 2, SAT serine acetyltransferase, TMEM161B Transmembrane Protein 161B, TNPO1 Transportin 1, YAP yes-associated protein.

Currently used GPX4 inhibitors contain RSL3, ML210, FIN56 as well as some different pharmacological inhibitor (DPI) compounds (Fig. 4a), inhibiting GPX4 functions via binding to or degrading GPX4 on its active site and show good anticancer activity [198, 199].

A study showed that HSP27 was able to up-regulate GPX4, resulting in stem cells in EC cells that could survive ferroptosis, which in turn led to bad prognosis within EC patients [200]. Western blot examination has revealed that the upregulated expression of DNAJ (Heat Shock Protein Family Member) B6 (DNAJB6) inhibited GPX4, promoted ferroptosis, and achieved anticancer effects [9]. Another study proposed that the neuropeptide LGI1 receptor A Disintegrin And Metalloproteinase Domain 23 (ADAM23) may cause ferroptosis in ESCC cells due to depletion of GPX4, SLC3A2, and SLC7A11 [201]. Shishido et al. experiments confirmed that 5-Aminolevulinic acid (5-ALA) inhibited the GPX4 system as well as upregulated HO-1 within ESCC tissues. It also promoted ferroptosis in ESCC, indicating its great antitumor effect [202]. Revealed by RNA immuno-precipitation assays, OIP5-AS1 binds to GPX4, whereas knockdown of OIP5-AS2 is able to downregulate GPX4 which in turn inhibits EC cell proliferation [203].

In the highly mesenchymal state, tumor cell relies on GPX4 to live, they also have higher sensitivity with GPX4 inhibitors [198]. Thus, the capacity of GPX4 suppressors in overcoming drug resistance is typically paired with targeted treatments as well as radiation treatments. However, combinations of multiple drugs tend to bring more side effects. Hangauer suggested that administering targeted therapy or drug-therapy either before or after GPX4 inhibitor could serve as an alternative approach to enhance therapeutic effect and mitigate toxic side effects, thereby offering a novel method for cancer treatment.

### Targeting NRF2 in EC

NRF2 act as a crucial regulator of antioxidant response, Its functions encompass the regulation of iron metabolism, management of exogenous substances, breakdown of reactive aldehydes and the synthesis of GSH and the regeneration of NADPH (Fig. 4b) [81]. In biopsy specimens of ESCC, negative expression of NRF2 predicts a good response to chemoradiotherapy as well as a better prognosis [204]. However, when ESCC is overexpressed, it predicts a poor prognosis for patients [205]. Immunohistochemistry showed that NRF2 expression in ESCC tissues and cells was much higher than normal [206]. Notably, NRF2 can activate Ca2 + /calmodulin-dependent protein kinase IIα (CaMKIIα), promote autophagy, and reduce the sensitivity of ESCC to radiotherapy [205].

Polygalacin D is a type of Chinese herbal extract, which inhibited cancer development through miR-142-5p/NRF2 axis in mouse model [207]. Liensinine was also able to suppress NRF2 expression and promote cellular ferroptosis to suppress ESCC progression [206, 208]. In ESCC patients, high NRF2 expression is associated with reduced PFS, OS as well as unfavorable therapeutic outcomes [209]. The aforementioned findings demonstrated that NRF2 level is positively linked to ESCC development.

NRF2’s expression degree profoundly influences tumor cells’ susceptibility to ferroptosis. Elevated NRF2 expression enables tumor cells to withstand ferroptosis, whereas decreasing its levels heightens cellular susceptibility to ferroptosis-inducing agents [210]. Biochemical purification results showed ARF as an important NRF2 regulator. It has been shown that NRF2-ARF interaction is active in P53 independent ferroptosis [211].

Several related studies have also shown that NRF2 has similar manifestations regarding other tumor cells, for example, high expression promoting tumor growth, metastasis and developing drug resistance [212–215]. In addition to its own role, GPX4 as well as SLC7A11 are also controlled by NRF2 [216]. Nevertheless, the exact mechanisms of genes in NRF2 that contribute to the anti-ferroptosis effect of ESCC remain to be identified.

### Targeting P53 in EC

P53 gene is an important tumor suppress gene, which mutated or inactivated in more than 50% tumors. In EC, p53 mutation is particularly prevalent, affecting roughly 40-60% of patients [217]. P53 inhibits cancer cell progression mainly by inhibiting cell cycle and promoting senescence and apoptosis. Research has unveiled that p53 can modulate ferroptosis in tumor cells through its influence on redox balance and metabolic pathways [218].

P53 regulates ferroptosis relatively. On one hand, P53 has the ability to activate the occurrence of ferroptosis, for example, P53 functions in inducing ferroptosis in tumor cell via inhibiting SLC7A11 transcription thereby downregulating cystine uptake [219]. Missense mutations in P53, like R273H, R175H, block NRF2-regulated SLC7A11 upregulation and repress its expression [220, 221]. P53 also targets spermidine/spermine N-acetyltransferase 1 (SAT1), which is related to polyamine metabolism, to modulate ferroptosis sensitivity [222]. Recently, it was found that radiation induced P53 activation, suppressed SLC7A11, triggered lipid peroxidation as well as ferroptosis within EC cells (Fig. 4c) [223].

In addition, P53 is able to suppress ferroptosis as well. For instance, in certain scenarios, the absence of p53 suppresses the nuclear accumulation of dipeptidyl peptidase-4 (DPP4), fosters the formation of DPP4 and NOX1 complexes, and thereby intensifies lipid peroxidation, leading to ferroptosis [65]. Therefore, P53’s functions on ferroptosis are complex and dependent on the type of mutation and the environment in which it is located, and its exact mechanism regulating ferroptosis in EC cells requires further investigation.

### Targeting Hippo‑YAP in EC

The Hippo-YAP signaling pathway modulates genes expression thereby affecting cellular morphology, density and adhesion properties. Dysfunction of this pathway is often associated with the development of SCC, including ESCC [224]. YAP, a key transcription agent located in the downstream of the Hippo pathway, functions in cell progression, generation and apoptosis [224].

In ESCC, YAP is frequently overexpressed, relating to histological stage or grade of the tumor. High levels of YAP often indicate poor OS and PFS [225]. Zhao indicated that downregulation of YAP strongly suppressed EC cell metastasis in vitro and in vivo (Fig. 4d) [226]. Research has indicated that ferroptosis relies on cell density and the Hippo signaling pathway, which is activated by the tumor suppressive gene NF2 [33]. Activation of NF2 can promote the downregulation of E3 ubiquitin ligase CRL4^DCAF1^, suppress Lats1/2 degradation in the hippocampal pathway, thereby promote YAP phosphorylation [227].

The expression of TFRC and ACSL4 mediated by YAP effectively regulates sensitivity to ferroptosis [33]. High expression of PARK2 promotes the degradation of YAP, thereby inhibits Hippo-YAP signaling pathway then achieves the effect of inhibiting tumor progression [228]. Thus, regulation of the Hippo-YAP pathway exhibits the potential to regulate ferroptosis in EC and then inhibit cancer progression.

### Targeting DNAJB6 in EC

DNAJB6 is a member of the HSP40/DNAJ partner family [229, 230]. It has multiple effects in diseases, for instance, it commonly accumulates in neurological diseases [231]. DNAJB6 is also observed to overexpress in ovarian tumor tissue, possibly a potential target for patient prognosis [232]. Another study showed that DNAJB6a inhibited the development of breast cancer cells [233].

At present, multiple investigations have been conducted regarding the function of DNAJB6 in EC. It is mentioned that DNAJB6a in EC cells can inhibit the generation of cancer cells through AKT1 (Fig. 4e) [234]. Jiang et al. revealed that typical features of ferroptosis were observed and GPX4 levels decreased in DNAJB6a-overexpressing ESCC cells. They therefore indicated that the elevated level of DNAJB6 induced ferroptosis. Also, patients with low levels of DNAJB6 are prone to suffer from lymphatic metastasis in comparation with high levels [9]. Nevertheless, the precise mechanism by which DNAJB6 induces intracellular iron-related ferroptosis still requires further investigation.

### Targeting SLC7A11 in EC

Existing studies suggest that SLC7A11 is a potential cancer therapeutic target [235]. An ideal anticancer therapeutic target should meet specific selectivity for cancer growth, exert toxic killing effects in cancer cells, and have few adverse effects in normal tissue cells, and SLC7A11 appears to meet these criteria [236]. Specifically, in cancer cells, oxidative stress is at high levels and cells in turn have higher protect requirement. Thus, tumor cell depends more on SLC7A11 in acquiring cysteine and keep redox homeostasis than normal tissues [235].

Several researches indicated that SLC7A11 can inhibit ferroptosis intracellular and promote drug resistance and radiation resistance [237–239]. High levels of SLC7A11 are associated with short PFS, OS as well as unsatisfactory treatment outcomes in ESCC patients (Fig. 4f) [209].

Currently, a number of compounds are recognized as inhibitors of SLC7A11, containing the ferroptosis-inducing agent erastin, sulfasalazine, cisplatin, sorafenib, and artesunate [199, 240, 241]. Sulfasalazine curbed the proliferation and colony-forming ability of EC cells, with effects escalating as the dosage increased [242]. Cisplatin has demonstrated substantial efficacy against advanced non-surgical EC in treatment settings [243, 244]. Guo proposed that combining erastin and cisplatin can promote the anticancer function. They suggest that cisplatin induces ferroptosis primarily through direct intracellular depletion of GSH [245], similar to the conclusions of Roh et al. [246]. In summary, SLC7A11 may be one target with great potential for EC treatment, especially survival outcomes and prognosis, however, highly effective specific drugs used in clinical practice are still lacking.

### Targeting competing endogenous RNAs (ceRNAs) in EC

Recently, ceRNAs has been studied, mainly to explore the role of mutual regulation between RNAs. Its regulatory network contains microRNAs (miRNAs), mRNAs, long non-coding RNAs (lncRNAs), and circulatory RNAs (circRNAs). MiRNAs can compete with mRNAs for miRNA response effectors (MREs) located on them and silence them [247]. CeRNAs containing MREs (mainly lncRNAs and circRNAs) control the expression and function of gene through competitively binding to miRNAs [247].

Meanwhile, ceRNAs contributed significantly in targeted therapy for cancer [248, 249]. MiR-27a is able to target KRAS to suppress the development of ESCC (Fig. 4h) [250], and its down-regulation reverses multidrug resistance in ESCC [251]. The expression of miR-27a-3p is upregulated when P53 is knockout, while TP53 status is associated with susceptibility to ferroptosis [252]. CircBCAR3 (hascirc0007624) overexpress in EC and is able to sponge miR-27a-3p to upregulate transporter-1, ultimately leading to proliferation, metastasis, invasion and ferroptosis of EC cells [253]. Lu’s team demonstrated that TMEM161B-AS1 could phagocytose miR-27a-3p and up-regulate GCH1 levels, leading to EC progression, suggesting that miR-27a-3p and TMEM161B-AS1 can further regulate the ferroptosis through affecting the GCH1 pathway [254].

As mentioned earlier, in ESCC, ADAM23 was demonstrated to be capable of inducing ferroptosis. Chen’s team put forward a ceRNA regulatory model for ADAM23: ARHGEF26-AS1 boosts ADAM23 expression via targeting the miR-372-3p/ADAM23 pathway, which in turn promotes ferroptosis [201]. Yao proposed that circPVT1 can target miR-30a-5p/FZD3 axis and accelerate tumor development. Specifically, circPVT1 showed remarkably high expression in ESCC cells which have the resistance to 5-fluorouracil, while after knocking-down, GPX4 and SLC7A11 were significantly down-regulated, promoting ferroptosis [255].

Other studies mentioned that lncRNA BBOX1-AS1 is able to mediate miR-513a-3p to regulate the SLC7A11 pathway in ESCC cells. Inhibiting this axis may also offer novel therapeutic avenues for EC. Circ0120816/miR-1305 has some potential in the treatment of ESCC by regulating GSH [256]. The study of ceRNAs is one of the hotspots today, interfering with any part of it results in hindering the onset of ferroptosis in EC. Exploring ceRNAs mechanisms related to ferroptosis has led to the creation of various innovative approaches for targeted esophageal tumor therapy.

### Targeting diterpenoids‑induced ferroptosis in EC

Diterpenoids, a class of compounds extracted from plants, have multiple pharmacological functions such as anti-oxidant and bactericidal. Several of them were found to exhibit anti-cancer functions on endothelial cells in laboratory settings [257].

Oridonin, a commonly utilized diterpenoid, has garnered significant interests due to anti-tumor properties [258]. It could induce apoptosis and deplete GSH in hepatic stellate cells [259]. Zhang revealed that EC cells treated with oridonin exhibited lipid peroxidation, development arrest and demise (Fig. 4g), Which is reversed by ferroptosis suppressors, the same phenomenon occurs in hepatic stellate cells [260]. They concluded that oridonin promoted ferroptosis in cells through regulating γ-glutamate cycle, which in turn achieved antitumor effects [261]. However, its clinical role remains to be further investigated.

Brusatol, another diterpenoid compound, was able to inhibit EAC development through affecting NRF2. Significant lipid peroxidation and ferroptosis were induced when brusatol was administered alone or in combination with cisplatin [262].

## The role of chemoradiotherapy in ferroptosis on EC

A significant proportion of EC patients are already in advanced stage at the time of detection and have associated local or distant metastases. For such inoperable patients, neoadjuvant concurrent chemoradiotherapy with or without surgery is mainly used [263]. Recently, neoadjuvant immunotherapy gave better survival expectations, current researches on compounds and interventions focusing ferroptosis in EC was summarized in a table (Table 1). However, many patients still fail to achieve the desired therapeutic effect [264].

**Table 1:** Current researches on compounds and interventions focusing ferroptosis in EC.

Tumor type	Compounds/interventions	Regulatory mechanism	Effect in tumor cell ferroptosis	Reference
EC	ML210	Irreversibly bind to the active selenocysteine site of GPX4	Activate	[91]
	RSL3	Irreversibly bind to the active selenocysteine site of GPX4	Activate	[27]
	FIN56	Promote the degradation of GPX4 protein	Activate	[30]
	PdPT	Reduce GPX4 intracellular abundance	Activate	[92]
	ARF	Inhibit NRF2 pathway	Activate	[210]
	TMEM161B-AS1	Phagocytose miR-27a-3p and up-regulate GCH1	Inhibit	[254]
	CircBCAR3	Sponge miR-27a-3p to upregulate transporter-1 (TNPO1)	Inhibit	[253]
	NF2	Downregulate CRL4 and activate YAP pathway	Inhibit	[227]
	Eprenetapopt	Deplete GSH	Activate	[271]
	MF-438	Target SCD1 then produce MUFAs and oleic acid	Activate	[268]
	Ionizing radiation	Activate PTGS2, NOX, ACSL4, P53, suppressed SLC7A11	Activate	[223, 238]
	DNAJB6	Inhibit GPX4 pathway	Activate	[9]
	Cisplatin	Inhibit SLC7A11, intracellular depletion of GSH	Activate	[245]
	Erastin	Inhibit system xc^-^ and reduce the cystine uptake	Activate	[11]
	Oridonin	Affecting the γ-glutamate cycle	Activate	[261]
EAC	Brusatol	Inhibit NRF2 pathway	Activate	[261]
ESCC	PARK2	Promotes the degradation of YAP	Activate	[228]
	Polygalacin D	Downregulate NRF2 through miR-142-5p and upregulate ROS	Activate	[207]
	Circ0120816	Inhibit miR-1305, which target TXNRD1	Inhibit	[256]
	CircPVT1	Target the miR-30a-5p/FZD3 axis	Inhibit	[255]
	MiR-27a	Target KRAS	Inhibit	[250, 251]
	5-ALA	Suppressed GPX4 and overexpressed HO-1	Activate	[202]
	Liensinine	Suppress NRF2 expression	Activate	[208]
	HSP27	Downregulate p53, upregulate GPX4	Inhibit	[200]

*5-ALA* 5-Aminolevulinic acid, *ACSL4* Long-chain-fatty-acid—CoA ligase 4, *CRL3* Cullin-RING E3 ligases, *DNAJB6* DnaJ Heat Shock Protein Family (Hsp40) Member B6, *EAC* esophageal adeno carcinoma, *EC* esophageal cancer, *ESCC* esophageal squamous cell carcinoma, *FZD3* Frizzled Class Receptor 3, *GCH1* GTP cyclohydroxylase 1, *GPX4* Glutathione Peroxidase 4, *HO-1* heme oxygenase-1, *HSP27* Heat shock protein 27, *NRF2* Nuclear factor erythroid derived 2-like 2, *PARK2* Parkinson disease protein 2, *PdPT* palladium pyrithione complex, *PTGS2* prostaglandin-endoperoxide synthase 2, *SCD1* Stearoyl-CoA Desaturase 1, *TXNRD1* thioredoxin reductase 1, *YAP* yes-associated protein.

The main influencing factors are multidrug resistance to chemotherapy and resistance to radiotherapy [265]. Recent research has indicated that radiotherapy or chemotherapy induces ferroptosis to some extent, which in turn enhances the sensitivity of patients to radiotherapy and obtains a good therapeutic effect (Fig. 4i). Alterations of ROS levels in cells induce the sensitivity of tumor cell with multidrug resistance to some drugs [266, 267]. Lei indicated that ionizing radiation (IR) was able to induce ferroptosis marker gene PTGS2. In parallel, the expression of 4-HNE significantly increased in FLO-1 cells following IR treatment [238]. They therefore concluded that IR was able to induce lipid peroxidation while increasing EC cells susceptibility to ferroptosis [238].

On the other hand, Luo demonstrated that MF-438 can target SCD1, promoting the sensitivity to radiotherapy, thereby strongly improves radiation therapy effect [268]. As mentioned earlier, SCD1 inhibits ferroptosis by producing MUFAs and oleic acid. The combination of the TP53 inhibitor Eprenetapopt with conventional anticancer agents has a better therapeutic effect [269, 270]. Fujihara found a substantial reduction in GSH levels in EC cell line OACM5.1 treated with eprenetapopt after untargeted metabolomics and label-free quantitative proteomics analysis [271]. It is therefore proposed that eprenetapopt induces ferroptosis by depleting GSH, which in turn limits tumor development [271]. Furthermore, the concurrent use of glycine and eprenetapopt effectively restrained the progression of EC and enhanced OS among patients [271].

A variety of conventional anticancer medications are capable of facilitating ferroptosis via multiple pathways, such as inhibiting system xc^-^, consuming GSH, and regulating GPX4 enzyme activity. Currently, inducing ferroptosis can curb tumor growth and significantly enhance the prognosis for EC patients. Additionally, ferroptosis has the potential to enhance EC susceptibility to radiochemotherapy.

## The role of immunotherapy in ferroptosis on EC

Immunotherapy is a research hotspot of oncology. Recently, significant advancements have been achieved in applying immunotherapy to patients with EC. The regimen of pembrolizumab in combination with chemotherapy is approved to be first-line treatment strategy of advanced EC [272]. The latest phase III RCT trial ESCORT-NEO showed a significant pCR advantage of neoadjuvant camrelizumab in combination with chemotherapy [273].

At present, neoadjuvant immunotherapy combined with concurrent chemoradiotherapy has also reached preliminary conclusions, showing further survival advantages and therapeutic effects [274]. Immune checkpoint inhibitors (ICI), which are central to immunotherapy, have significantly influenced the strategy treating multiple cancers including EC. The KEYNOTE-180 trial and ATTRACTION-1 trial have demonstrated that ICI pembrolizumab and nivolumab are effective to metastatic EC [272, 275].

Ferroptosis was first reported to induce immunogenic cell death in vitro and in vivo by Efimova et al. The release of DAMPs, especially ATP and high mobility group box 1 (HMGB1), promotes ferroptotic immunogenic cell death, thereby suppresses the development of tumor cells [110]. In EC patients, the integration of immunotherapy and ferroptosis offers novel approaches for personalized treatment. Lu creatively developed a prognostic model for EC patients based on immune-related FRGs, thereby greatly link immunotherapy with ferroptosis [276]. They discovered that patients with EC in the high-risk category derived greater benefit from ICIs [276].

In patients with EC, ICDs appear to induce antitumor effects. Zhang’s team combined various cell death signals to classify patients into different subtypes [277]. Transcriptomic versus proteomic profiling suggests that signaling of ferroptosis positively correlates with immune activation in ESCC [277]. Activating the immunogenic properties of siderophilic tumor cells could pave the way for identifying new treatment targets, offering a potential treatment approach for EC patients [277]. Further perspective, ferroptosis may help coordinate immunotherapy with chemoradiation in EC patients. Therefore, further research is necessary to confirm, investigate, and ultimately implement these findings in clinical settings.

## Prediction of the prognosis utility of ferroptosis in EC

Zhu detected FRGs within EAC patients according to the TCGA database, they revealed that FRGs are primarily concentrated in lipid metabolism, paste metabolism, energy metabolism, and antioxidant metabolism. Four ferroptosis-related genes (FRG) (CARS1, GCLM, GLS2, and EMC2) were identified according to COX regression analysis, which can bring value to the OS prognosis of EAC patients. Among them, GLS2, CARS1, and EMC2 were able to induce ferroptosis. Moreover, they were positively related to prognosis. GCLM, additionally, acts to inhibit ferroptosis [278].

Notably, Lu’s team has identified 45 FRGs through analyzing aberrant gene expression within ESCC, they subsequently constructed a predictive model based on COX regression analysis. Their findings indicated that less risk scores are associated with having more CD4^+^ memory active T cells, CD8^+^T cells, and macrophages. In addition, their screened genes such as SCP2, MAPK, and PRKAA1 were proved to implicated in multiple aspects of cellular ferroptosis. Further investigation led them to designate ALOX12, ALOX12B, ANGPTL7, DRD4, MAPK9, SLC38A1, and ZNF419 as prognostic FRGs in ESCC patients. Specifically, ALOX12, ANGPTL7, DRD4, and MAPK9 expression levels were notably reduced, whereas that of SLC38A1 and ZNF419 were greatly elevated. In contrast, ALOX12B level remains unchanged [276].

Following their results, Song et al. chose SLC38A1, which exhibited significant expression differences, to delve into the influence on the proliferation and spread of ESCC cells. Their study ultimately revealed that SLC38A1 promotes the proliferation and spread of ESCC cell [279]. Zhao identified 6 FRGs with prognostic value in 117 ESCC samples [280]. Among those, the expression of PRNP, SLC3A2, SLC39A8, and SLC39A14 was negatively correlated with the prognosis of ESCC patients, while the expression of ATP6V0A1 and LCN2 was the opposite. They also assessed infiltration of immune system, finding that the expression of ATP6V0A1, SLC39A14, SLC39A8, and LCN2 positively related to B cells infiltrating activities. However, SLC3A2 and PRNP expression correlated negatively.

Liu identified 18 pairs of differentially expressed ferritin-associated lncRNAs through the analysis of tumor tissues or samples, then subsequently developed a prognostic signature model [281]. After analysis using statistic methods, he concluded that survival expectancy, immunotherapy efficacy, and drug sensitivity could be predicted in EC patients, this would help making characteristic therapy strategies [281].

Many aforementioned FRGs hold distinct prognostic significance for EC treatment, with some serving as independent prognosis indicators. Delving deeper into and refining our understanding of them can offer early indications of EC development, thus aiding in the timely implementation of preventive and therapeutic strategies in clinical settings. Moreover, these prognostically significant FRGs are closely linked to the TME. In addition, when integrating the correlation of ICIs, this strong association is leveraged to clinically make more treatment regimens, thereby unlocking the potential of prognostic FRGs in EC immunotherapy.

## Conclusion

Ferroptosis, as a non-apoptotic RCD regulated by iron, is generated and carried out under stringent molecular regulatory mechanisms. Although many studies have confirmed the close relationship between ferroptosis and cancer, the study of ferroptosis in EC is still in its infancy. Currently, it is mainly focused on screening FRGs using genetic data from databases, and these FRGs can serve as prognostic indicators, acting as protective or risk factors in EC patients. For patients with EC, it is very important for the early diagnosis of EC since the early stages EC’s prognosis is good.

For patients with advanced disease, surgery is often not feasible; combined chemoradiotherapy and immunotherapy have become a better treatment. However, insensitivity to radiotherapy and resistance to chemotherapy often lead to unsatisfactory treatment outcomes. Also, there are limitations in the scope of adaptation of immunotherapy. Ferroptosis brings new directions for the treatment of EC. Many FRGs have been identified as prognostic genes in EC.

There has been considerable research into how ferroptosis works in EC cells, studies have revealed that ferroptosis are able to render EC cells more responsive to chemotherapy, lower their resistance to radiation, and broaden the scope of EC immunotherapy. Specifically, FRGs and immune infiltrates are closely related, and many key genes during ferroptosis are also immune-related. Certain ferroptosis inducing agents in combination with chemotherapeutic agents demonstrate greater effectiveness compared with either agent alone and improve sensitivity to chemotherapeutic agents.

However, ferroptosis is a mixed blessing; we should fully investigate the potential toxic side effects of inhibitors or inducers of key pathways of ferroptosis and strive to achieve specific triggering of ferroptosis in cancer cells while avoiding adverse effects for healthy cells. Most ferroptosis inducers currently used target SLC7A11 or GPX4. Most of the ferroptosis inducers with agreement from FDA are not included in standard therapy strategies guidelines of EC. Others, such as RSL3, are banned for use in clinical trials because of pharmacokinetic reasons, thereby can only be used for laboratory studies.

New approaches such as nanomedicine approaches, photodynamic therapy may be effective in solving these problems. Nanoparticles (NPs) offer versatile and customizable platforms that improve the solubility, permeability, and bioavailability of therapeutic agents. In addition, NPs exhibit favorable pharmacokinetic properties, enable sustained and controlled drug release, and allow selective targeting of specific cells, tissues, or organs-collectively enhancing the therapeutic efficacy of conventional drugs. Also, by lowering systemic toxicity, nanocarriers enable dosage reductions in chemotherapeutic drugs without sacrificing effectiveness [282]. For instance, biocompatible cerium oxide nanoparticles (CeO_2_ NPs) exhibited potent SOD and catalase activities, efficiently scavenging multiple free radicals and lipid peroxidation products both intracellularly and extracellularly. These activities effectively prevented or alleviated ferroptosis in RSL3-induced cells [283].

Also, photodynamic therapy (PDT) has emerged as a promising approach to cancer therapy, leveraging cytotoxic ROS to eliminate cancer cells [284]. Some well-designed agents like PpIX-1-DG, a new chimeric peptide, have great potential in cooperation with nanotherapeutics in various aspects including Smart drug delivery systems that activate in response to tumor-specific signals and include real-time monitoring to regulate ferroptosis [285]. These approaches also have great potential in personalized cancer treatment.

APR-246 (also called eprenetapopt) is a newly identified ferroptosis inducer which is in clinical development with a focus on TP53 mutation. The recent studies have shown that the expression of SLC7A11, the cystine/glutamate transporter, was identified as a superior determinant of response to APR-246 [286]. To be specific, eprenetapopt depletes cellular antioxidant glutathione levels by increasing its turnover, triggering ferroptosis [271]. As discussed above, eprenetapopt was closely related to EC ferroptosis, providing potential applications for EC patients. Other ferroptosis inducers with clinical trial include Imidazole Ketone Erastin (IKE), which functions in inhibiting the SLC7A11, has been shown to play an important role in the treatment of ferroptosis in some cancers [287, 288], future studies could examine this as a possible target of EC ferroptosis.

Sorafenib, an FDA-approved ferroptosis inducer for hepatocellular carcinoma, demonstrated great treatment effect in inhibiting SLC7A11 [289]. Past studies have found that sorafenib triggers antiproliferative and pro-apoptotic signals in human esophageal adenocarcinoma cells [290], and Tiam1 siRNA can enhance the sensitivity of sorafenib on esophageal squamous cell carcinoma in vivo [291]. While recent studies revealed that it also functions in EC ferroptosis.

Chen kai et al. have found that knockdown Apoc1 can overcome resistance of sorafenib in EC cells and promoted erastin and sorafenib induced ferroptosis by upregulating the levels of ROS and downregulating the level of GSH [292]. Xiying Yu used sorafenib as a tumor promoter to establish an NMBzA-induced rat ESCC carcinogenesis model, the multi-omic result of their research indicated that NMBzA-induced rat ESCCs are accompanied by progressive hyperactivations of the FAT-Hippo-YAP1 axis, and inhibitors of YAP1 block the growth of rat ESCCs [293]. As mentioned before, the Hippo-YAP1 axis regulates the sensitivity of ferroptosis through affecting the expression of TFRC and ACSL4. In combination, sorafenib may also be of great research value in EC ferroptosis.

Some other studies, including CeRNA, have shown promising effects; however, the precise mechanism still requires further investigation and cannot be directly applied in clinical practice. Therefore, future studies should identify valuable FRGs from bioinformatics data, conduct in-depth mechanistic studies, investigate the mechanisms of function in EC from multiple omics, and perform empirical proof to further develop corresponding ferroptosis inhibitors or inducers.

At the same time, ferroptosis combined with immunotherapy and chemoradiotherapy has great potential; it could be crucial for harmonizing immunotherapy and chemoradiotherapy. For resistant patients, the application of ferroptosis for treatment will bring new hope. Therefore, in-depth studies should be conducted in combination with ferroptosis and immune checkpoint therapy as well as chemo/radiotherapy. We cannot neglect that EC as well as other cancers, are of great heterogeneity, having great demands of personalized treatment, which will become a focal point in further studies. The emergence of ferroptosis brings great help to provide personalized treatment for EC patients. There remain great uncertainties in the fundamental research and clinical application of ferroptosis. Future studies will undoubtedly offer substantial benefits to EC patients.

## Supplementary information

abbreviation list
