# Supplementary material for: Exploring the role of ferroptosis in esophageal cancer: mechanisms and therapeutic implications
Source: Cell Death Discov. 2025 Aug 25;11:405. doi: 10.1038/s41420-025-02696-2 (PMC12379297; doi:10.1038/s41420-025-02696-2)
Supplement: Supplementary file 1 — abbreviation list [file 41420_2025_2696_MOESM1_ESM.docx]

**Supplementary material legends:** A list contrasting the abbreviations with their corresponding full names. The abbreviations are defined in the text at first use.

**Abbreviations–definitions:**

AA–arachidonic acid

ACACA – acetyl-CoA carboxylase α

ACSL4 – Acyl-CoA synthetase long-chain family member 4

AdA–adrenic acid

ADAM23 – A Disintegrin And Metalloproteinase Domain 23

AMPK – AMP-activated protein kinase

ALOX – Arachidonate lipoxygenase

ALI – Acute lung injury

ARE – Antioxidant response element

ATP – Adenosine triphosphate

BH4 – Tetrahydrobiopterin

CAF – Cancer-associated fibroblast

CaMKIIα – Ca2+/calmodulin-dependent protein kinase IIα

ceRNA – Competing endogenous RNA

CH – Clonal haematopoiesis

CoQ10 – Ubiquinone-10 (coenzyme Q10)

CRL4^DCAF1^ – Cullin-RING ligase 4-DCAF1

Cys – cysteine

Cys2 – cystine

DAMP – Damage-associated molecular pattern

DC – Dendritic cell

DHODH – Dihydro-orotate dehydrogenase

DNAJB6 – DnaJ heat-shock-protein family member B6

DPP4 – Dipeptidyl-peptidase-4

EC – esophageal cancer

EAC – esophageal adenocarcinoma

EMT – Epithelial-to-mesenchymal transition

ER – Endoplasmic reticulum

ESC – Embryonic stem cell / esophageal squamous cell (context-dependent)

ESCC – esophageal squamous cell carcinoma

ETC – Electron-transport chain

FIN – Ferroptosis inducer (e.g., FIN56)

FRG – ferroptosis-related genes

FSP1 – Ferroptosis-suppressor protein 1 (AIFM2)

GCH1 – GTP-cyclohydrolase 1

GCL – glutamate-cysteine ligase

GGT5 – γ-glutamyltransferase

GLS – Glutaminase

Gln – glutamine

GPX4 – Glutathione peroxidase 4

GSH – Glutathione (reduced)

GSSG – Glutathione disulphide (oxidised glutathione)

H_2_O_2_ – hydrogen peroxide

HCAR1 – Hydroxy-carboxylate receptor 1

HCT – Haematopoietic cell transplantation

HIF – Hypoxia-inducible factor

HMGB1 – High-mobility group box 1

HNE (4-HNE) – 4-Hydroxynonenal

HSP – Heat-shock protein

ICD – Immunogenic cell death

ICI – Immune-checkpoint inhibitor

IFN-γ – Interferon-gamma

IKE – Imidazole Ketone Erastin

iNOS – nitric oxide synthase 2

IR – Ionising radiation

IRF1 – interferon regulatory factor 1

KEAP1 – Kelch-like ECH-associated protein 1

LDH – Lactate dehydrogenase

LIP – Labile iron pool

lncRNA – Long non-coding RNA

LO· – lipid hydroxyl radicals

LOO· – lipid peroxyl radicals

LOOH – Lipid hydroperoxide

LPCAT3 – lysophosphatidylcholine acyltransferase 3

MAPK – Mitogen-activated protein kinase

MBOAT – Membrane-bound O-acyl-transferase

MCT1 – monocarboxylate transporter 1

MDS – Myelodysplastic syndrome

MDSC – Myeloid-derived suppressor cell

MGUS – Monoclonal gammopathy of undetermined significance

MPO – Myeloperoxidase

MREs – miRNA response effectors

MUFAs – Monounsaturated fatty acids

MZB – marginal zone B cells

NCOA4 – Nuclear receptor coactivator 4

NF2 – Neurofibromin-2 (merlin)

NGS – Next-generation sequencing

NPs – Nanoparticles

NK – Natural-killer (cell)

NOX – NADPH oxidase

NRF2 – Nuclear factor erythroid-2 related factor 2

O_2_ – oxygen

O_2_^−^ – superoxide radicals

OAA – Oxalo-acetic acid (contextually GOT1)

OX-LDL – Oxidised low-density lipoprotein

OXPHOS – Oxidative phosphorylation

PDAC – Pancreatic ductal adenocarcinoma

PDT – photodynamic therapy

PE – phosphatidylethanolamines

PEBP1 – Phosphatidylethanolamine-binding protein 1

PHLDA2 – pleckstrin homology domain family A member 2

PMN – polymorphonuclear

PNH – Paroxysmal nocturnal haemoglobinuria

POR – Cytochrome P450 oxidoreductase

PPAR – Peroxisome proliferator-activated receptor

PUFA – Polyunsaturated fatty acid

RCD – Regulated cell death

ROS – Reactive oxygen species

RSL3 – RAS-selective lethal 3

RTA – radical trapping antioxidant

SAT1 – spermidine/spermine N-acetyltransferase 1

SCD1 – Stearoyl-CoA desaturase 1

SFA – saturated fatty acids

SLC – Solute carrier family member (e.g., SLC7A11)

SNAT – Sodium-coupled neutral amino-acid transporter

SOD – Superoxide dismutase

STAT – Signal transducer and activator of transcription

STEAP – Six-Transmembrane Epithelial Antigen of Prostate

TAM, TANK – Tumour-associated macrophage, NK cell

TCA – tricarboxylic acid

TF – Transferrin

TFRC – Transferrin receptor

Th17 – T-helper-17 cell

TIME – Tumor immune microenvironment

TMEM161B-AS1 – Transmembrane protein 161B antisense RNA 1

TME – Tumour micro-environment

Treg – Regulatory T cell

TXNRD – thioredoxin reductase

UBE1A/UBA1 – Ubiquitin-like modifier activating enzyme 1

VDAC – Voltage-dependent anion channel

VTE – Venous thrombo-embolism

WTAP – Wilms’ tumour 1-associating protein

YAP – Yes-associated protein

·OH – hydroxyl radical

α-KG – α-ketoglutarate

5-ALA – 5-Aminolevulinic acid
